# Contribution of CNS and extra-CNS infections to neurodegeneration: a narrative review

**DOI:** 10.1186/s12974-024-03139-y

**Published:** 2024-06-06

**Authors:** Pinja Kettunen, Jari Koistinaho, Taisia Rolova

**Affiliations:** Helsinki Institute of Life Science, University of Helsinki, Helsinki, Finland

**Keywords:** Neuroinflammation, CNS infection, Neurodegeneration, Brain infections, Alzheimer’s disease, Parkinson’s disease, Amyotrophic lateral sclerosis, Multiple sclerosis

## Abstract

Central nervous system infections have been suggested as a possible cause for neurodegenerative diseases, particularly sporadic cases. They trigger neuroinflammation which is considered integrally involved in neurodegenerative processes. In this review, we will look at data linking a variety of viral, bacterial, fungal, and protozoan infections to Alzheimer’s disease, Parkinson’s disease, amyotrophic lateral sclerosis, multiple sclerosis and unspecified dementia. This narrative review aims to bring together a broad range of data currently supporting the involvement of central nervous system infections in the development of neurodegenerative diseases. The idea that no single pathogen or pathogen group is responsible for neurodegenerative diseases will be discussed. Instead, we suggest that a wide range of susceptibility factors may make individuals differentially vulnerable to different infectious pathogens and subsequent pathologies.

## Background

Central nervous system (CNS) infections have been suggested to act as a possible trigger for neurodegenerative diseases such as Alzheimer’s disease (AD), Parkinson’s disease (PD), amyotrophic lateral sclerosis (ALS) and multiple sclerosis (MS). They are particularly interesting when trying to explain the high prevalence of sporadic neurodegenerative diseases which genetic factors alone are unable to explain.

For a while, the field of neurodegeneration has been dominated by the idea that neurodegenerative diseases are caused by the pathological accumulation of toxic aggregating proteins such as amyloid-β (AD), α-synuclein (PD). However, the processes that trigger protein accumulation in various brain areas are still poorly understood, apart from rare familial cases where the accumulation is caused by dominantly inherited mutations. Since research targeting aggregating proteins has been limited in its ability to produce effective treatments, many researchers have turned towards complementary hypotheses [1].

The ‘infection hypothesis’ suggests that infections can push the system to pathology by direct harm caused by the infection or by triggering neuroinflammation [1–3]. As we will later see, microbes can harm the host organism in multiple ways, such as via toxic molecules, inhibition of host immune function, disruption of tissue tight junctions, induction of proteotoxic stress, or by directly killing host cells. On the other hand, they trigger an immune response which can be detrimental to host cells (‘bystander effect’) especially if the inflammatory state becomes chronic. However, not all infections lead to neurodegeneration, and genetic susceptibility factors may help explain why some people are more vulnerable to the detrimental effects of infections than others. Furthermore, infections are only one possible cause for neuroinflammation alongside other factors such as traumatic injury, ischemic injury, autoimmunity, metabolic disease, and lifestyle (diet, sleep, exercise, stress, smoking) which we will not cover here.

To assess whether infections are involved in neurodegeneration, we need to determine (a) whether patients suffering from neurodegeneration have a history of CNS infections, (b) whether non-CNS infections can cause neurodegeneration without direct CNS invasion, (c) whether infections increase risk of neurodegeneration, and (d) whether infections are able to induce neuroinflammation and neurodegeneration-related changes at the cellular and tissue level, possibly even after infection clearance. It would also be important to know whether pathogen infections and known risk factors for neurodegenerative diseases interact, and whether eradicating infections is an effective treatment and/or prevention strategy for neurodegenerative diseases. Since aggregating proteins are integral to neurodegenerative diseases, we will look at how aggregating proteins and infections could fit into the same picture.

## Main text

### Prevalence of CNS infections in the global population

Neurological symptoms such as fatigue, headache, sensory changes, cognitive changes, psychosis, seizures, paresis, and coma are common symptoms of many infectious diseases [4]. For example, around 20–30% of all Covid-19 patients have been reported to have neurological symptoms such as fatigue or cognitive impairment even months after acute respiratory infection [5]. It is often difficult to know whether the neurological symptoms are secondary to the systemic disease state or caused by direct infection of the CNS. However, a variety of pathogens from viruses and bacteria to fungi and protozoa have been detected in the CNS using histochemical and molecular biology methods [6]. In fact, many globally significant diseases, such as malaria, borreliosis/Lyme disease, HIV, diphtheria, tuberculosis, candidiasis, and syphilis have neurological presentations where the otherwise systemic infection enters the brain [4].

Differences in target tissues, infection routes, replication, and release patterns are likely to affect the outcome of the infection [7]. Some CNS-infiltrating pathogens specifically target brain cells such as neurons or glia. For example, rabies virus, Zika virus, tick-borne encephalitis virus and herpes viruses specifically target neurons. They often cause severe disturbance of the CNS homeostasis, such as encephalitis or meningitis, which can cause death or disability. Such severe infections are associated with many disorders including neurodegeneration in survivors [8–12].

Other pathogens are more likely to enter the CNS during disturbance such as immunodeficiency, inflammation, blood–brain barrier (BBB) breakdown, trauma, neurosurgical procedures or other infections. Such pathogens include *Candida* yeasts*, Cryptococcus* and *Mycorales* fungi, the protozoan parasite *Toxoplasma gondii,* as well as the bacteria *Streptococcus pneumoniae, Staphylococcus aureus* and *Mycoplasma* sp. [13]. These microbes can cause encephalitis in immunocompromised patients, but such severe representations are rare in otherwise healthy individuals.

Certain pathogens, such as *Helicobacter pylori*, periodontal bacteria, and gut microbes, have been linked to neuropathology without a direct infection of the CNS. In fact, changes of the gut-brain axis, such as gut dysbiosis (imbalance of the gut microbiome), are very common in patients of neurodegenerative diseases [14]. It is thought that systemic inflammation caused by extra-CNS infections can jump across the CNS barriers (most notably the BBB) via proinflammatory factors such as cytokines, extracellular vesicles, and small lipid mediators, which triggers neuroinflammation [15–20]. Interestingly, this jump can occur prior to BBB disruption [21] which suggests that systemic infections could disturb BBB function from both sides. Furthermore, changes in the gut-brain axis can affect the CNS also via the secretion of neurotransmitters, vitamins, important fatty acids such as butyrate, and amyloid proteins by gut microbes [14].

Finally, most neurodegenerative disease patients do not have a history of severe CNS pathologies such as encephalitis or meningitis. Thus, common pathogens that cause milder infections have gained the interest of scientists. For example, most of us carry latent infections (Table 1) which means that such infections could potentially explain common diseases such as dementias. Latent infections are characterized by alternating active and quiescent periods, where the pathogens hide from the immune system only to reactivate under favorable conditions such as stress-induced immunosuppression. To do this, they use immunomodulatory molecules [22, 23], biofilms [24], cysts [25], encapsulation [23], and integration to the host genome (viruses) [26]. While the reactivation of latent infections can lead to severe pathology such as encephalitis [11], majority of these infections are mild or asymptomatic. However, the chronic presence of these pathogens in the CNS is thought to cause long lasting or reoccurring neuroinflammation [25, 27, 28]. In fact, latent infections have been linked to severe conditions such as cancer [29–31] and neurodegenerative diseases.

**Table 1:** Examples of common pathogens that cause chronic latent infections and their prevalence in different adult human populations

Virus	Abbreviation	Associated disease	Immunoglobulin G prevalence, adult (females, males or pooled)	Known to enter the CNS	Refs.
Human herpesviruses					
Herpes simplex virus 1/Human herpesvirus 1	HSV-1	Cold sores, encephalitis	UK: 71%, 69% (female, male) Latin America & the Caribbean: 88% (pooled) Middle East & North Africa: 92% (pooled) Sub-Saharan Africa: 96% (pooled) Asia: 77% (pooled) East Asia (Japan): 63%, 55% (female, male)	Yes	[32–36]
Herpes simplex virus 2/Human herpesvirus 2	HSV-2	Genital herpes	Europe: 11%, 5% (females, males) Eastern Mediterranean: 8%, 3% (females, males) UK: 17%, 15% (females, males) Americas: 24%, 12% (females, males) Latin America & Caribbean: 25%, 14% (females, males) Africa: 44%, 25% (females, males) South-East Asia: 10%, 7% (females, males) East Asia (Japan): 7%, 9% (females, males) Western Pacific: 15%, 7% (females, males)	Yes	[32, 33, 37, 38]
Varicella zoster virus/Human herpesvirus 3	VZV, HHV-3	Chicken pox, Varicella, Shingles, Herpes zoster	UK: 91%, 94% (females, males) Western Europe (Netherlands): 95%, 93% (females, males) US: 98% (pooled) Middle East (Iran): 78% (pooled) East Asia (South Korea): 93% (males)	Yes	[32, 39–42]
Epstein-Barr virus/Human herpesvirus 4	EBV, HHV-4	Mononucleosis	UK: 96%, 93% (females, males) US: 89% (pooled) Middle East (Qatar): 100%, 96% (females, males) Sub-Saharan Africa (Nigeria): 20% (pooled) East Asia (China): 90% (pooled, > 8-year-olds) Australia: 89%, 96% (females, males)	Yes	[32, 43–46]
Cytomegalovirus/Human herpesvirus 5	CMV, HHV-5		Europe: 45–95%, 35–44% (females, males) UK: 59%, 96% (females, males) North America: 24–81%, 48% (females, males) Latin America: 58–94% (females) Africa: 55–97% (pooled) East Asia (Japan): 60% (females)	Yes	[32, 47, 48]
Human (beta)herpesvirus 6	HHV-6		UK: 91%, 90% (females, males) Northern Europe (Finland): 86% (pooled) Western Europe (France): 76% (females) US: 98% (pooled) Middle East (Qatar): 93%, 71% (females, males) South America (Brazil, Ecuador): 91–92%, 90% (females, males) Northern Africa (Morocco): 20% (females) Sub-Saharan Africa: 60–90% (females) East Asia (Japan): 79% (pooled)	Yes	[32, 49–53]
Human (beta)herpesvirus 7	HHV-7		UK: 96%, 93% (females, males) Central America (Mexico): 98% (pooled) South Africa: 99% (pooled) East Asia (Japan): 44% (pooled) Germany/Israel/Poland/Australia/US: 75–85% (pooled)	Yes	[32, 54, 55]
Kaposi’s sarcoma-associated herpesvirus/Human herpesvirus 8	HHV-8		UK: 8%, 9% (females, males) Southern Europe (Spain): 7%, 6% (females, males) Middle East (Iran): 15% (females) US: 3–5% (pooled) Sub-Saharan Africa: 40% (pooled) The Caribbean: 3% (pooled) South-East Asia: 4% (pooled)	No	[32, 56–60]
Human papillomaviruses					
Human papillomaviruses	HPV	Anogenital cancers	Among asymptomatic individuals: Africa: 21–23%, 17% (females, males) Eastern Africa: 35%, 38% (females, males) North America: 5–14%, 9–45% (females, males) South America: 14–17%, 9–34% (females, males) Central America: 21%, 26% (females, males) Europe: 7–10%, 7–31% (females, males) Eastern Europe: 29% Asia: 8–11%, 3% (females, males) Eastern Asia: 19%, 15% (females, males)	No	[61–64]
Parasites					
*Toxoplasma gondii*		Toxoplasmosis	Europe: 30% (females) Northern Europe (Finland): 14%, 20% (females, males) UK: 27%, 29% (females, males) Eastern Europe (Serbia): 45%, 55% (females, males) Eastern Mediterranean: 40% (females) USA & Canada: < 10% (females) Latin America: 45% (females) Africa: 31–40%, 30% (females, males) South-East Asia: 25% (females) Western Pacific: 11% (females)	Yes	[32, 65–69]

### General mechanisms of microbe-induced pathology in the brain

Potential mechanisms for microbial neuropathology are diverse. Many of them are secondary to the microbe’s attempt to infect, survive and replicate inside the host. For example, many viruses and intracellular parasites lyse the host cell as part of their reproductive cycle to release the newly produced pathogens. However, there are other ways in which pathogens are harmful. For example, many molecules that microbes use to survive can be toxic to the host [70]. Furthermore, the infiltration of pathogens into host tissues via pore formation [71], modulation of adhesion proteins [72], and hijacking of the host endocytosis [73] can disrupt important host cell-to-cell contacts [74, 75], cause the fusion of adjacent cells (syncytia) [75–78], and cause leakage of molecules across host barriers such as the BBB [75].

Similarly, by hijacking the host protein synthesis machinery, cytoskeleton, and intracellular transport system, the pathogen facilitates its own replication and spread. Simultaneously, it can also disrupt the homeostatic function of the host cell, such as normal protein synthesis and axonal transport [79, 80]. This can lead to problems such as proteotoxic stress [81]— a common occurrence in neurodegenerative diseases [82].

Furthermore, the immunomodulatory and immunosuppressive tactics utilized by pathogens to evade the host immune system [22, 23, 83] can lead to secondary infections, or to the disruption of homeostatic functions performed by the immune cells, e.g. waste clearance performed by microglia.

Finally, host immune response to pathogen surface structures or secreted products can become as detrimental as the infection itself. For example, cytokines, antimicrobial molecules, reactive oxygen species (ROS), nitric oxide, phagocytosis, and forced cell death are used by immune cells to destroy pathogens [84]. These same mechanisms can be harmful to nearby host cells if there is no balance between optimal pathogen clearance and excessive inflammatory response [75, 84].

### A brief introduction of discussed neurodegenerative diseases

#### Dementia (unspecified)

World Health Organization estimates that around 55 million people are affected by dementia around the world and the number is thought to increase to 139 million by 2050 [85]. Dementia itself is not a disease but a syndrome (a collection of symptoms) which describes the decline of cognitive abilities such as memory, learning, concentration, planning, motivation, language processing, reasoning, and thinking. It also affects mood and can cause anxiety, depression, or aggression. The most common causes for dementia are AD (70% of all cases), frontotemporal dementia, vascular dementia, and dementia with Lewy bodies. All of them have slightly different main symptoms and disease dynamics, but the overlap between diseases is considerable. Determining which disease causes dementia in each patient is difficult and often based on symptoms alone. Thus, many studies handle dementia as a collective unit.

#### Alzheimer’s disease

AD causes around 60–70% of all dementia cases [85]. It is characterized by progressive degeneration of the brain parenchyma and the accumulation of proteinaceous amyloid-β and tau inclusions. The pathology is associated with neuroinflammation, glial activation, and dysfunction of the BBB and the brain blood circulation.

Apolipoprotein E (APOE) ξ4 allele is the most prevalent genetic risk factor for late-onset AD (begins after the age of 65). It increases the risk of AD ~ threefold in heterozygotes and ~ 15-fold in homozygotes [86]. APOE is a lipid carrier molecule that facilitates the trafficking of cholesterol and phospholipids between cells. Due to its effect on lipid membranes, APOE is involved in many cellular functions, including the regulation of immune cells such as microglia [87, 88].

Many other risk mutations for AD are enriched in microglia, including mutations in TREM2, ABI3, ABCA7, CD33, and CR1 [89–91]. For example, triggering receptor expressed on myeloid cells 2 (TREM2) regulates microglial functions such as phagocytosis, microglial activation, and inflammatory responses, which are crucial for CNS immunity. Rare mutants of TREM2, such as the single nucleotide mutation R47H, have been associated with increased risk of AD and other dementias [92, 93].

#### Parkinson’s disease

PD is the second most common neurodegenerative disease after AD. In 2019 it was estimated to have affected over 8.5 million individuals globally [94]. PD is the most common cause of parkinsonism, a motor disorder characterized by tremors, rigidity, bradykinesia, and posture changes. PD also causes a variety of gastrointestinal (GI) symptoms. The symptoms are a result of the progressive loss of dopaminergic neurons of substantia nigra. The process is associated with neuroinflammation and the accumulation of amyloid-like α-synuclein.

Many important PD risk genes are involved in endosomal (*LRRK2*, *SNCA*) and lysosomal (*GBA, TMEM175, CTSB*) function, mitophagy (*PINK1*, *PARK2*), autophagy (*SNCA, KAT8*), RNA processing (*TARDBP*), and antigen presenting (*HLA-DRB6*, *HLA-DQA1*) [95]. Many of these functions are important for immune response. Thus, mutations in these pathways can make an individual susceptible to the negative effects associated with infections. For example, the PD risk mutation p.G2019S in the LRRK2 gene lead to higher reovirus mortality in mutant mice compared to wildtype animals. The effect seems to be caused by heightened inflammatory response [96, 97]. Interestingly, the LRRK2 p.G2019S mice were better at controlling septic *Salmonella typhimurium* infection [96] which hints at a pathogen-specific effect.

#### Amyotrophic lateral sclerosis (ALS) and ALS-like syndromes

Amyotrophic lateral sclerosis, also known as Lou Gehrig’s Disease, is a fatal motor neuron disease characterized by progressive degeneration of upper and lower motor neurons. It leads to muscle weakness and the loss of motor control, which leads to death when it spreads to respiratory muscles. Intracellular aggregation of proteins such as transitive response DNA-binding protein 43 (TDP-43), fused in sarcoma (FUS) and superoxide dismutase 1 (SOD1) are common.

Noncoding hexanucleotide GGGGCC repeat expansion in C9orf72 is the most common genetic mutation found in ALS patients (40–50% of familial cases, 5–10% of sporadic cases) and frontotemporal dementia [98]. The gene is involved in many cellular functions including lysosomal function, stress granule formation and immune function. Several gain-of-function and loss-of-function mechanisms have been suggested to explain how mutations in C9orf72 increase ALS risk [99, 100].

Several mutations in SOD1 explain around 2% of all ALS cases and 20% of familial cases. SOD1 is an important antioxidant enzyme that protects cells from ROS, which is secreted by immune cells during pathogen infections [101]. A combination of infection and genetic susceptibility to ROS could make an individual vulnerable to neurodegeneration [102].

#### Multiple sclerosis

MS is the most common inflammatory demyelinating disease in the brain and spinal cord. It affects around 2.8 million people worldwide [103]. It is characterized by defects in sensory, motor, and autonomic functions. The most common clinical course involves alternating relapses and remissions where flare-ups of symptoms are followed by periods of full or partial recovery. Common histopathological findings include focal demyelinated plaques or lesions surrounded by activated T-cells and myeloid cells such as microglia. The leading theory is that MS is caused by autoimmunity against host myelin-producing cells. In fact, many MS risk genes are involved in autoimmunity, such as the human leukocyte antigen class II allele HLA-DBR1*15:01 and the interleukin 2 receptor subunit alpha (IL2RA) [104]. The possible role of infections in MS remains unknown. On the one hand, microbial molecular mimics of myelin-associated proteins such as the myelin basic protein (MBP) could trigger autoimmunity [105, 106]. On the other hand, it has been suggested that excessive hygiene in modern societies leads to deficient training of immune cells which can cause them to attack host tissues (‘the hygiene hypothesis’).

### Role of pathogens in neurodegenerative diseases

Many CNS-infiltrating pathogens have been found in neurodegenerative disease patients using histological and PCR-based methods. Many of them show positive associations with neurodegeneration in population level studies (often around 1.3–3.0-fold increase in risk, see Table 2). The strength of these associations is of the same magnitude as other commonly accepted neurodegeneration-associated factors such as cardiovascular disease, stroke, and sleep disorders [107–112].

**Table 2:** An overview of studies investigating the association between herpesviruses and dementia, AD, PD, ALS, or MS

Disease	Herpesvirus	Comparison	OR/HR/RR, 95% CI, p-value	Conclusion/Additional info	Cohort	Refs.
Dementia	HSV-1	Seropositive vs. Seronegative	aHR 1.18, 95% CI: 0.83–1.68	No association between dementia and HSV-1. HSV-1 increased the risk of decline in global cognition	Rotterdam Study	[113]
Dementia	VZV	Herpes zoster CNS infection vs. no infection	HR 1.94, 95% CI: 0.78–4.80	No association between herpes zoster and dementia	Danish nationwide	[114]
Dementia	1. HSV-1 2. VZV	Infection vs. No infection	1. aHR 1.18, 95% CI: 1.16–1.20, p < 0.001 2. aHR 1.09, 95% CI: 1.07–1.11, p < 0.001	Mild association between dementia and HSV-1 or VZV	South Korea National Health Insurance Service	[115]
Dementia	Pooled	Persistent herpesvirus infection vs. no infection	aHR 2.35, 95% CI: 1.38–3.98	Persistent herpesvirus infection increased the risk of dementia	3 Finnish cohorts UK biobank	[116]
Dementia	VZV	Herpes zoster patients vs. control	aHR 1.12, 95% CI: 1.05–1.19	Herpes zoster mildly increased the risk of dementia	South Korea National Health Insurance Service	[117]
Dementia	VZV	1. Herpes zoster patients vs. control 2. Antivirals vs. no treatment in herpes zoster patients	1. aHR 1.11, 95% CI: 1.04–1.17 2. HR 0.55, 95% CI: 0.4–0.77	Herpes zoster mildly increased the risk of dementia Antiviral therapy reduced the risk of dementia in herpes zoster patients	Taiwan National Health Insurance Research Database	[118]
Cognitive decline	1. CMV 2. EBV	Seropositive vs. Seronegative	1. HR 1.74, 95% CI: 0.12–15.0 2. HR 2.24, 95% CI: 0.36–16.4	No association between cognitive decline and CMV or EBV	Finnish Health 2000 Survey	[119]
Dementia	CMV	Positive vs. negative 1. Age 40–59 2. Age 60–79 3. Age > 80	1. OR 11.7, 95% CI: 2.5–49.4 2. OR 1.8, 95% CI: 1.1–3.2 3. OR 1.3, 95% CI: 0.5–2.8	Association between CMV and dementia	Korea National Health Insurance Database	[120]
Cognitive decline	1. HSV-1 2. CMV 3. EBV 4. VZV	IgG positive vs. Negative	1. p = 0.018 2. p = 0.011 3. p = 0.128 4. p = 0.187	Poisson regression model supported the association between HSV-1, CMV and cognitive decline. No association with EBV or VZV	Baltimore ECA cohort	[121]
Cognitive decline	CMV	Seropositive vs. Seronegative	p = 0.425	No association between CMV and cognitive decline in individuals age 40–70 year using multivariate testing	UK Biobank	[122]
Alzheimer’s disease	HSV-1	Seropositive vs. Seronegative	aHR 1.13, 95% CI: 0.77–1.66	No association between HSV-1 and decline in global cognition	Rotterdam Study	[113]
Alzheimer’s disease	HSV-1	IgG and IgM positive vs. negative APOE ε4 carriers	aHR 3.68, 95% CI: 1.08–12.55, p = 0.04	Increased risk of AD in HSV-1 positive APOE ε4 carriers, but not in non-carriers	Bordeaux-3c prospective cohort	[123]
Alzheimer’s disease	pooled	Systemic antiherpetic drug vs. untreated	aHR 0.85, 95% CI: 0.75–0.96, p = 0.009	Systemic antiherpetic drugs reduced the risk of AD Included drugs: Aciclovir, Famciclovir, Valaciclovir, Valganciclovir	French medico-administrative database	[124]
Alzheimer’s disease	HSV-1	1. IgG positive + APOE ε4 vs. negative controls 2. IgG positive + risk score vs. negative controls	1. OR 4.55, 95% CI: 1.29–16.06, p = 0.02 2. OR 2.35, 95% CI: 1.21–4.56, p = 0.01	Increased risk of AD in HSV-1 positive APOE ε4 heterozygotes but not in APOE ε4 non-carriers. Risk also increased by a risk score that was calculated using nine genes: ABCA7, BIN1, CD33, CLU, CR1, EPHA1, MS4A4E, NECTIN2 and PICALM	Medical Biobank in Umeå	[125]
Alzheimer’s disease	1. CMV 2. HSV-1	Seropositive vs. Seronegative	1. RR 2.15, 95% CI: 1.42–3.27, p < 0.001 2. RR 0.84, 95% CI: 0.62–1.16, p < 0.001	Association between CMV and AD irrespective of race, HSV-1 status, or APOE ε4 status No association between HSV_1 and AD	Rush Memory and Aging Project, The Religious Orders Study,The Minority Aging Research Study	[126]
Parkinson’s disease	1. HSV 2. VZV	1. Infection vs. no infection 2. Herpes zoster vs. control	1. OR 0.79, 95% CI: 0.73–0.86, p < 0.001 2. OR 0.88, 95% CI: 0.83–0.92, p < 0.001	Inverse association between PD and HSV, and PD and VZV Antiherpetic treatment reduced the risk of PD. Included drugs: Acyclovir, Valacyclovir, Valganciclovir, Famciclovir, Ganciclovir, Penciclovir	Medicare beneficiaries	[127]
Parkinson’s disease	HSV-1	IgG titer with infection vs. no infection	p = 0.045	HSV-1 antibody prevalence and titer were slightly higher in’PD patients. CMV and HHV-6 prevalence or IgG levels not altered. Protective’AD protective mutation in PILRA was not associated with HSV-1 in’PD	IRCCS Santa Maria Nascente, Don Gnocchi Foundation	[128]
Parkinson’s disease	VZV	herpes zoster vs. Control	HR 1.50, 95% CI: 1.16–1.93	Association between PD and herpes zoster	Taiwan National Health Insurance Research Database	[129]
Parkinson’s disease	VZV	herpes zoster vs. Control	aHR 1.17, 95% CI: 1.1–1.25	Mild increase in PD risk in herpes zoster patients	Taiwan National Health Insurance Research Database	[130]
Parkinson’s disease	HSV-1	IgG titer in patients vs. healthy controls	p < 0.001	Increased level of HSV-1 antibodies in’PD patients	40 patients	[131]
Parkinson’s disease	HSV-1	IgG titer in patients vs. Healthy controls	NA	Increased level of antibodies against HSV-1 capsid, envelope, and excreted antigen in PD patients	52 patients	[132]
Parkinson’s disease	HSV-2	IgG titer in patients vs. healthy controls	p < 0.005 for capsid antibodies p < 0.001 for envelope and excreted antibodies	Increased level of HSV-1 antibodies in’PD patients. Total IgG level in CSF was within normal limits	56 patients	[133]
Multiple sclerosis	EBV	1. Infection vs. no infection 2. Seroconversion vs. seronegative	1. HR 26.5, 95% CI: 3.7–191.6, p = 0.001 2. HR 32.4, 95% CI: 4.3–245.3, p < 0.001	Association between EBV and MS. No association between MS and HSV-1, HSV-2, CMV, or VZV	Military personel	[134]
Multiple sclerosis	pooled	Herpesvirus infections vs. controls	OR 2.07, 95% CI: 1.8–2.37	Association between herpesvirus infections and MS	Meta-analysis of 245 datasets	[135]
Multiple sclerosis	HHV-8	Presense of HHV-8 genome in the blood of patients vs. controls	p = 0.0017	HHV-8 genome detected in 18.5% (10/54) of patients and 3% (4/130) of healthy controls	54 patients, 130 controls	[136]
Multiple sclerosis	HHV-7, HHV-8	Presense of viral material in patients vs. controls	No statistical tests done	HHV-7 and HHV-8 mRNA was detected in oligodendrocytes in some individuals by FISH but not by RT-PCR in post mortem brain tissues	6 patients, 3 controls	[137]
Amyotrophic lateral sclerosis	1. HHV-6 2. HHV-8	Seropositive vs. Seronegative	1. OR 3.2, p = 0.102 2. OR 8.4, p = 0.064	Association detected, sample size too small for statistical significance	20 ALS cases, 20 controls	[138]

Furthermore, in vitro cell culture studies and in vivo animal models have shown that many pathogens can induce neuroinflammation and neurodegeneration-related changes, such as glial activation, leukocyte infiltration, cytokine production, BBB dysfunction, and the accumulation of neurodegeneration-related aggregating proteins.

The diversity of pathogens that have been implicated in neurodegenerative diseases is striking, even though none of the connections have been conclusively proven. For example, Sipilä et al. screened the association of hospital-treated pathogen infections (925 International Classification of Diseases-10 codes) with dementia using the medical records of over 700 000 participants in Finnish and United Kingdom databanks. The dataset included pathogens of all classes: viruses, bacteria, fungi, and protozoan parasites, and both CNS and non-CNS infections. All types of infections increased the risk of dementia which suggests that there is no single pathogen or infection type that would be solely responsible for dementia. Furthermore, multiple simultaneous or subsequent infections increased the risk of dementia more than single infections (adjusted hazard ratio (aHR) 5.08 vs. 3.04 respectively), particularly in the case of combined virus and bacterial infections (aHR 8.15, 95% CI: 4.73–14.05) [116]. Similar results have since been published by Bohn et al. [139]. However, there may be disease-specific differences. For example, Fang et al. found no association between CNS infections and ALS in a study of 4000 ALS patients and 20 020 matched controls [140].

**Fig. 1:**
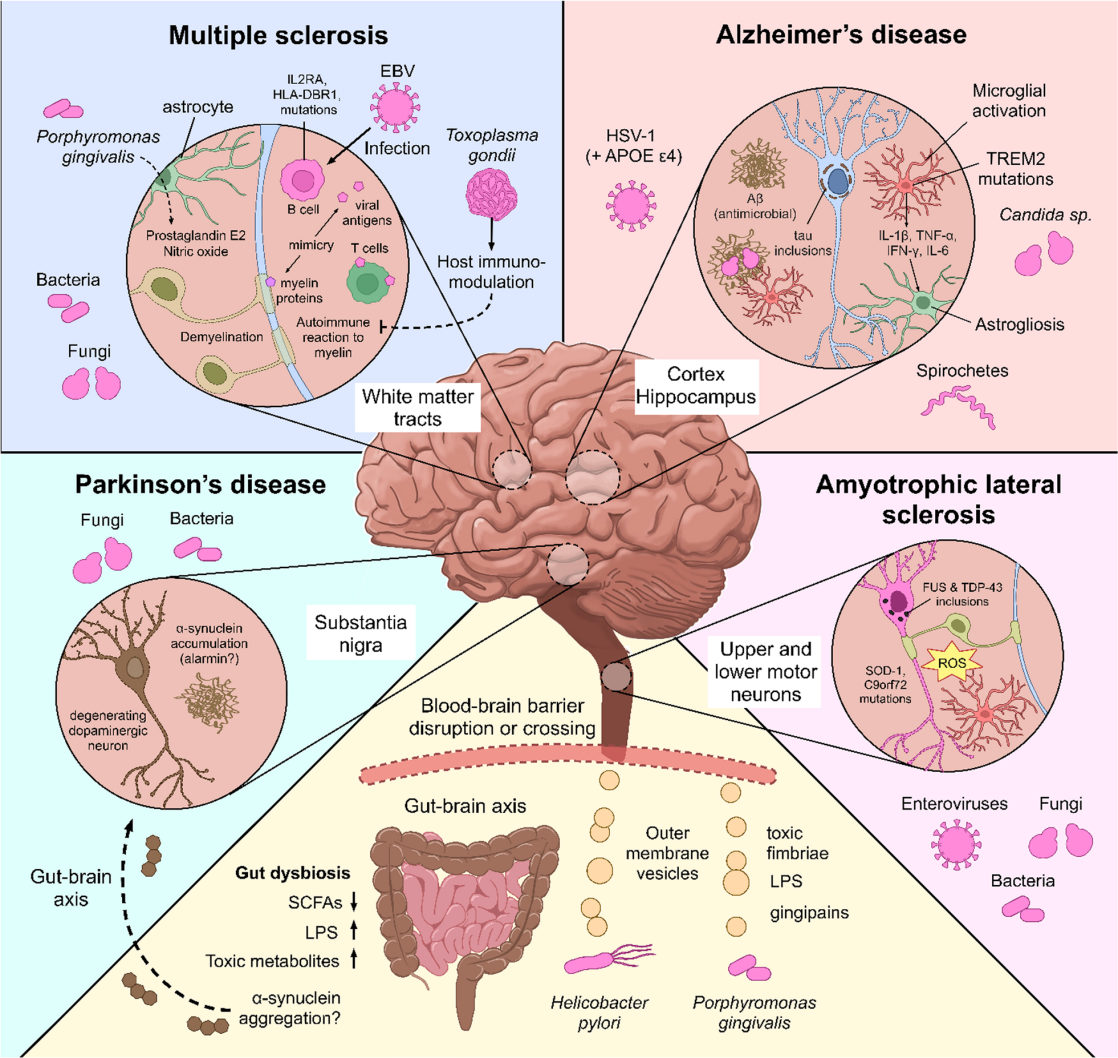
A variety of CNS infections are associated with AD, PD, ALS, and MS. *IL2RA* Interleukin-2 receptor subunit alpha

Below, we will continue exploring this diversity by reviewing the data that supports the involvement of CNS infections by herpesviruses, enteroviruses, HIV, SARS-CoV-2, spirochete bacteria, bacterial pneumonia, fungi, and *T. gondii* in unspecified dementia, AD, PD, ALS, and MS (Fig. 1). We will also briefly mention extra-CNS infections that have strong link to neurodegeneration, namely periodontal disease, *H. pylori* infections, and gut dysbiosis. Simultaneously, we will discuss the importance of individual susceptibility, which may explain why neurodegeneration is triggered in some individuals while most are left unaffected. The final section of this review discusses the idea that neurodegeneration-related aggregating proteins could function as innate immune proteins that bind microbes and repress their replication while activating antimicrobial immune pathways.

#### Herpesviruses

The family of human herpes viruses includes herpes simplex virus 1 and 2 (HSV-1 and HSV-2), varicella-zoster virus/human herpes virus 3 (VZV/HHV-3), Epstein-Barr virus/human herpes virus 4 (EBV/HHV-4), cytomegalovirus/human herpes virus 5 (CMV/HHV-5) and human herpes viruses 6, 7 and 8 (HHV-6, HHV-7, HHV-8). They cause common ailments such as cold sores of the mouth and genitals (HSV-1 and HSV-2), chickenpox/shingles (VZV), and mononucleosis/ ‘the kissing disease’ (EBV). HSV-1 is also the most common cause of encephalitis worldwide.

While many members of this family of viruses have been implicated in neurodegenerative diseases, it is unlikely that any of these viruses would be the sole cause of any neurodegenerative disease. After all, despite the extremely high prevalence of herpesviruses in the global population, most us are not affected by neurodegeneration.

Thus, it is not surprising that studies attempting to find correlation between neurodegenerative diseases and herpesviruses have yielded mixed results (Table 2). However, accounting for genetic risk factors associated with neurodegenerative diseases might help explain this discrepancy. For example, the association between APOE ε4 and HSV-1 in AD was first suggested by Ruth Itzhaki et al. in the late 1990s, when they reported that the odds of finding APOE ε4 allele in HSV-1 positive AD patients was almost 17 times higher compared to HSV-1 negative individuals without AD (OR 16.8, 95% CI: 3.61–77.8) [141].

Linard et al. and Lopatko Lindman et al. have also reported a 3.68–4.55-fold increase in AD risk in HSV-1 positive APOE ε4 carriers [123, 125]. Furthermore, Lopatko Lindman et al. reported an association between AD, HSV-1, and a risk score which was calculated from the presence of mutations in nine AD risk genes (ABCA7, BIN1, CD33, CLU, CR1, EPHA1, MS4A4E, NECTIN2, and PICALM) (OR 2.35, 95% CI: 1.21–4.56, P = 0.01) [125]. Interestingly, the combination of APOE ε4 with other pathogens, including HSV-2, did not increase AD risk [125]. It suggests that the genotype-microbe interactions are somewhat pathogen-specific.

The mechanisms through which genetic risk factors, such as APOE ε4 allele, increase AD risk, are still under investigation. However, it has been suggested that the APOE isoform may affect for example HSV-1 latency and frequency of reactivation [141–143] or the number of oral lesions [144].

Cell culture models also support the involvement of HSV-1 in AD*. *In vitro studies by Cairns et al., Qiao et al. and D’Aiuto et al. have shown that HSV-1 can induce AD-like pathology in human induced pluripotent stem cell (hiPSC) -derived neural cultures and 3D organoids[145–148]. The signs of AD pathology included changes in neuronal morphology, the formation of syncytia, neuronal loss, amyloid-β and tau accumulation, expression changes in amyloid-β processing genes (PSEN1, PSEN2, BACE1), increased expression of pro-inflammatory cytokines (TNF-α, IFN-γ, IL-1β, IL-6, IL-10, IL-4, CXC3R) as well as gliosis characterized by changed astrocyte morphology, increased expression of genes involved in astrogliosis (e.g. glial fibrillary acidic protein, GFAP) and microglial activation (CD11b, CD68, HLADR) [145–148]. Fruhwürth et al. have also reported that HSV-1 infection downregulates TREM2 pathway in hiPSC-derived microglia which leads to impaired interferon-β induction and impaired phagocytosis of HSV-1 infected neurons [149]. One possible mechanism for HSV-1-induced neuropathology is through the binding of the virus envelop glycoprotein D and the Aβ42 peptide. The amino acid residues involved in Aβ42-binding are left open, which means that the Aβ42-virus complex can act as a seed for Aβ oligomerization[150]. While this binding property may have evolved as part of the innate immune system (see later section), the process has likely become harmful to the host in conditions such as AD.

VZV is another herpesvirus, which has been researched in terms of neurodegeneration. Some studies have reported a mild increase in the risk of dementia (1.09–1.11-fold) [115, 117, 118] and PD (1.17–1.5-fold) [129, 130] in herpes zoster patients (detectable reactivation of VZV) while others have found either no association or an inverse association [114, 127, 151, 152]. Interestingly, Cairns et al. have reported that VZV induced gliosis and secretion of proinflammatory cytokines in hiPSC -derived neural cultures, but not the accumulation of amyloid-β or tau. Instead, VZV induced the reactivation of quiescent HSV-1 infection which then led to the accumulation of Aβ and phosphorylated tau [145]. Similar reactivation could be induced by other viruses such as SARS-CoV-2 [153–155].

Like HSV and VZV, EBV has been linked to many neurodegenerative diseases [156, 157]. The strongest link has been identified between EBV and MS. Up to 32-fold increase in MS risk has been reported following EBV infection [8, 134, 135]. Furthermore, MS is very rare in individuals who are seronegative for EBV [158]. EBV can infect neurons directly, disrupt BBB integrity, and cause neuroinflammation [156]. However, the prevailing theory is that EBV predisposes the host to MS-related autoimmunity through the infection of host B cells as well as through molecular mimicry of host myelin-associated proteins myelin basic protein (MBP) and glial cell adhesion molecule (GlialCAM) by the EBV nuclear antigen 1 (EBNA1) [105, 106, 159]. Since EBV is almost ubiquitously present in the population (90%), the affected individuals likely carry other vulnerabilities to autoimmune diseases. For example, the MS risk factor HLA-DRB1*15:01 can facilitate EBV entry into host B cells [160, 161]. Lifestyle factors, such as smoking, can further modulate this interaction [162]. Furthermore, another EBV protein, EBNA2, binds and alters the expression of MS-associated host genes which may increase MS risk. The effect is dependent on the presence of known protective or risk mutations for MS (listed in Table 3) [163].

**Table 3:** A list of publications that studied the interactions between infections and known risk genes for AD, PD, ALS, and MS

Disease	Gene mutation	Pathogen	Result	References
Alzheimer’s disease	APOE ε4	HSV-1	APOE ε4 allele is more common in HSV-1 carriers than in non-carriers	[141]
Alzheimer’s disease	APOE ε4	HSV-1	AD risk is increased in HSV-1 positive APOE ε4 carriers compared to non-carriers	[123]
Alzheimer’s disease	APOE ε4, Risk score genes: ABCA7, BIN1, CD33, CLU, CR1, EPHA1, MS4A4E, NECTIN2 and PICALM	HSV-1 HSV-2 CMV	AD risk is increased in HSV-1 positive APOE ε4 carriers compared to non-carriers. HSV-2 or CMV infection was not associated AD irrespective of APOE genotype. Genetic risk score calculated based on nine AD risk genes increased AD risk	[125]
Alzheimer’s disease	APOE ε4	HSV-1	Expression of latency-associated transcripts was slower in APOE ε4 mice compared to non-carriers	[142]
Alzheimer’s disease	APOE ε4	*Chlamydia pneumoniae*	Higher bacterial burden in AD patients who carry APOE ε4 compared to non-carriers	[164]
Parkison’s disease	LRRK2 p.G2019S	*Salmonella typhimurium*, reovirus	Mice carrying PD risk mutation LRRK2 p.G2019S controlled Salmonella typhimurium infection better than wildtype mice. However, they displayed higher mortality due to reovirus encephalitis, which was associated with increased markers of inflammation	[96]
Parkinson’s disease	LRRK2 R1441G	*Porphyromonas gingivalis*	Microglial activation and loss of dopaminergic neurons in LRRK2 R1441G mice after oral *P. gingivalis* administration. Increased TNF-α, IL-1β and α-synuclein and reduced zonula occludens-1 expression in the colon, increased serum interleukin 17 A (IL-17A), and increased IL-17 receptor A (IL-17RA) expression in the brain	[165]
ALS	SOD1 G85R	Coxsackievirus B3	Reduced lifespan and earlier start of motor dysfunction in SOD1 G85R mutation carriers compared to normal mice after coxsackievirus B3 infection	[166]
Multiple sclerosis	HLA-DBR1*15:01	EBV	MS risk allele HLA-DBR1*15:01 facilitates EBV entry into host B cells	[160, 161]
Multiple sclerosis	HLA-DBR1*09:01	EBV	HLA-DRB1*09:01 allele is associated with higher levels of blood biomarker for EBV reactivation (Zta-IgA) than non-carriers in healthy individuals. The effect was higher in smokers than non-smokers	[162]
Multiple sclerosis	SNPs near genes: Tumor necrosis factor receptor-associated factor 3 (TRAF3); Cluster of differentiation 40 (CD40); TNF Alpha Induced Protein 8 (TNFAIP8); TNF Receptor Superfamily Member 1A (TNFRSF1A); T-Box Transcription Factor 6 (TBX6); REST Corepressor 1 (RCOR1)	EBV	MS risk alleles and protective alleles alter the host interaction with EBV envelope protein EBNA2, which may affect EBV latency and MS risk	[163]

Antiherpetic drugs such as valacyclovir and acyclovir have been reported to reduce symptoms and slow disease progression in dementia patients. Linard et al. have reported that intake of at least one systemic antiherpetic drug reduced the risk of AD (aHR 0.85, 95% CI: 0.75–0.96, p = 0.009) in a cohort of 6642 subjects over the age of 65. Most subjects had undergone only a single intake of antiherpetic drugs and regular treatment was rare [124]. Antiviral therapy also reduced the risk of dementia in a Taiwanese population cohort study of 39,205 herpes zoster patients (HR 0.55, 95% CI 0.40–0.77) [118] and a South Korean cohort study of 34 505 herpes zoster patients (aHR 0.79, 95% CI: 0.69–0.90)[117]. The typical length of antiviral drug treatment was not specified for these studies. A study by Young-Xu et al. on 87 687 HSV-positive US veterans over the age of 50 years found that antiherpetic medication was associated with lower dementia risk when compared to the untreated group (aHR 0.75, 95% CI: 0.72–0.78). The reduction in dementia risk was associated with lower markers of neuroinflammation [167]. It is noteworthy that these therapies are not specific to any specific herpesvirus and that the positive effect could also be mediated by other viral infections.

In contrast, Schnier et al. found no convincing association between antiherpetic drug use and reduction in dementia risk in a study of 2.5 million individuals aged over 65 years. They only found a small and heterogenous negative effect in an analysis of four European databases from Wales, Germany, Scotland and Denmark. However, the typical length of antiherpetic drug treatment was relatively short: only 1–2 weeks [168]. In fact, it has been suggested that the antiherpetic/antiviral treatment lengths commonly administered in Europe are not long enough to show positive effects in these database-driven association studies. For example, the above-mentioned study by Young-Xu et al. reported that increasing length of antiviral treatment is associated with larger reductions in dementia risk in symptomatic HSV carriers. While any antiherpetic medication reduced the risk of dementia by 25% (HR = 0.75, 95% CI: 0.72–0.78), treatments longer than one year reduced the risk of dementia by 43% (HR = 0.57, 95% CI, 0.53–0.61). In the subgroup receiving medication for less than 30 days, the reduction was negligible (HR = 0.93, 95% CI: 0.87- 0.98) [167]. Thus, more studies testing the efficacy of longer antiherpetic drug treatments are needed. An ongoing clinical study by Columbia University [169] is currently assessing the efficacy of 7–8-week valacyclovir treatment in HSV-1 and HSV-2-positivie patients suffering from mild AD [170].

#### Enteroviruses

Enteroviruses, such as poliovirus, coxsackievirus, echovirus, enterovirus-A71, and enterovirus-D68 have gained interest in the field of motor neuron diseases due to their ability to infect motor neurons [171]. For example, poliovirus, the most famous enterovirus, attacks motor neurons of the spinal cord and brain stem causing neuroinflammation which can lead to irreversible paralysis (poliomyelitis aka. Polio). Around 28% of poliomyelitis survivors develop motor neuron disease (post-polio syndrome) decades after acute disease suggesting a chronic or reactivated infection [172].This disorder resembles ALS because it is characterized by gradual weakening and atrophy of specific muscles (often the limbs affected by poliomyelitis years earlier) due to the loss of motor neurons in the brainstem and spinal cord. Other symptoms include muscle fasciculations, fatigue, pain, sleep disturbance, and sometimes problems breathing or swallowing. However, post-polio syndrome can often be distinguished by the life history of poliomyelitis as well as slower progression and more generalized fatigue [173].

The relationship between ALS and ALS-like syndromes is still unclear, as is the involvement of enteroviruses in ALS. Some studies have found enteroviral RNA in ALS and motor neuron disease patients more frequently than in controls using PCR methods [174–177]. In contrast, others have failed to detect enterovirus RNA in the spinal cord of ALS patients [178–180]. The discrepancies could be explained by geographical differences (positive results in France, UK, Japan vs. negative in US and Australia) or by random variation introduced by small sample sizes (< 30 samples per group for all studies except one positive study where the number of samples was ten times higher).

Interestingly, Xue et al. have reported that sublethal coxsackievirus B3 infection can cause an increase in proinflammatory gene expression, TDP-43 pathology, neuronal damage, and immune cell infiltration in the CNS of normal C57BL/6J mice. In addition, coxsackievirus B3-infected mice carrying an ALS-related mutation SOD1^G85R^ displayed also a reduction in their lifespan and earlier start of motor dysfunction than non-infected ALS mice. Their results suggest that while the virus alone is able to cause neurodegenerative changes in these mice, a genetic susceptibility is needed for the onset of motor dysfunction [166].

In contrast, MS is a disease which affects the myelin sheaths around axons instead of the neurons themselves. Based on the limited evidence available, enteroviruses are not involved in the development of MS. For example, Kuusisto et al. found no evidence of enterovirus infection in the serum or cerebrospinal fluid of 17 MS patients [181]. Similarly, Perlejewski et al. detected enterovirus by RT-qPCR in the cerebrospinal fluid of only one MS patient (1 out of 34) [182].

#### HIV

HIV/AIDS is an infectious viral disease that compromises the host immune system due to the selective tropism of the virus to immune cells such as CD4 positive T helper cells, macrophages, dendritic cells, and microglia. The replication cycle of HIV kills the host cell which results in depletion of host immune cells and generalized immune deficiency. If left untreated, the patients succumb to secondary infections typically within 10 years [183]. In fact, HIV patients are susceptible to other opportunistic infections such as candidiasis, toxoplasmosis and bacterial pneumonia [184–186] as well as the reactivation of other latent infections such as herpesvirus infections [187, 188].

Interestingly, HIV has been linked to multiple neurodegenerative diseases. Around half of HIV/AIDS patients develop HIV-associated neurocognitive disorder (HAND) even with effective antiretroviral medication [189, 190]. Many of the symptoms resemble dementia, such as difficulties in learning, memory, decision making, and concentration. Patients also display neuroinflammation, neuronal loss, microglia/macrophage activation, multinucleated giant cells, and diffuse atrophy of many brain areas [191–193]. According to Wendelken et al. HAND-associated brain atrophy is exacerbated by the AD risk factor APOE ε4 [194]. Furthermore, neuropathologies such as progressive brain atrophy and microglia/macrophage activation, are present even in those with successful antiviral therapy which suggests that the pathology is mainly driven by neuroinflammation [195–197]. However, low-level viral replication in the CNS cannot be completely discounted even with successful suppression of the viral load in the plasma [191]. Interestingly, the use of antiviral therapy in HIV positive individuals is associated with an increase in the CSF levels of Aβ40 and Aβ42 compared to untreated HIV patients. Patients suffering from HIV-associated dementia (HAD) display reduced CSF levels of Aβ40 and Aβ42 compared to neurocognitively unimpaired individuals. The former is also commonly observed in AD patients [198]. These results suggest that HIV impairs Aβ clearance to the CSF, which may be a result of increased Aβ deposition into mature plaques. This process may be exaggerated in HAD patients. Increased CSF Aβ40 and Aβ42 levels following antiviral therapy would then indicate rescued Aβ clearance, which is however not enough to rescue cognitive functions [199].

Parkinsonism is another possible, albeit rare, outcome of HIV infection. There are multiple possible causes for parkinsonism in people living with HIV, including secondary infections, HAND, dopamine-blocking drugs such as neuroleptics, adverse reaction to antiretroviral therapy, and HIV encephalitis. Thus, the mechanistic connection between HIV and parkinsonism is not clear-cut. In general, the emergence of antiretroviral therapy has reduced the occurence of HIV-associated parkinsonism. Amelioration of symptoms following antiretroviral treatment, or the discontinuation of dopamine-blocking drugs, has also been reported [200]. Hence, HIV testing is worth considering in patients with otherwise unexplained parkinsonism.

Several case studies have also reported ALS-like syndrome in HIV patients, including in vivo signs of upper and lower motor neuron involvement [201–211]. However, the authors of this article are unaware whether motor neuron loss has been documented in post mortem or not. The patients often display earlier onset than in classical sporadic ALS [209]. The causation between HIV and ALS-like syndrome is yet unproven, and the putative mechanism is still unknown. However, the mechanism is likely indirect since HIV specifically targets immune cells such as microglia (instead of motor neurons). Similar to HIV and parkinsonism, antiretroviral therapy has been an effective treatment in many cases of HIV-associated ALS-like syndrome [202, 204, 208, 209] which suggests that the possibility of HIV infection should be taken into consideration in patients at high risk of exposure. In contrast, reduced risk of MS and reduced rate of relapsing have been reported in HIV positive individuals. It is currently not known whether the reduction in MS risk is a result of the HIV infection itself or secondary to antiretroviral therapy. Multiple explanations have been suggested. On the one hand, the depletion of CD4 + T cells by HIV could inhibit the development of CD4 + T cell-associated autoimmunity in untreated or late stage HIV patients. On the other hand, antiretroviral therapy could also inhibit other CNS viruses than HIV, including EBV, which could subsequently reduce MS risk. So far, the results have been variable and clear conclusions cannot be drawn. This and many other open questions in the field of HIV and MS have recently been reviewed by Stefanou et al. [212].

#### SARS-CoV-2

Severe acute respiratory syndrome coronavirus 2 (SARS-CoV-2) is a respiratory virus which caused the recent COVID-19 pandemic. Interestingly, CNS symptoms are common during and after SARS-CoV-2 infections, and they can last for extended periods of time (‘long covid’). A meta-analysis by Ceban et al. reported that twelve or more weeks after COVID-19 diagnosis 32% of individuals suffered from fatigue (68 included studies), and 22% of cognitive impairments (43 included studies) [5]. In a study by Xu et al. the burden of neurologic sequelae was 70 per 1000 persons after one year [213]. Changes in brain anatomy, such as reduction in grey matter thickness and global brain volume, have also been detected in COVID-19 patients – even milder cases [214]. Microhaemorrhages are also common [215–217].

This has triggered the questions whether SARS-CoV-2 can infect the brain, and whether the COVID-19 pandemic could increase the prevalence of neurodegenerative diseases in the future. Particularly, AD has been implicated due to the cognitive changes seen during and after COVID-19 disease. The diseases also share the risk factor APOE ε4, which increases the risk of late onset AD as well as severe COVID-19 disease [217, 218]. Furthermore, severe COVID-19 disease and mortality due to it are more common in AD patients.

To answer the first question: Post mortem studies have detected the presence of viral components in neurons, glia, and brain endothelial cells in deceased COVID-19 patients (the cases were not selected based on neurological symptoms) [219–223]. Consistently, stem cell-derived 2D and 3D models have shown that SARS-CoV-2 can infect the choroid plexus epithelium, astrocytes, subset of neurons, and possibly even microglia [224–234]. Thus, CNS infection by SARS-CoV-2 is possible.

Signs of neuropathology are also present after SARS-CoV-2 infection. Histopathological signs of neuroinflammation such as astrogliosis, microglial activation [219, 220, 235], BBB disturbance [235, 236], and infiltration of peripheral immune cells [219, 235] have been reported in COVID-19 patients. Stem cell studies have also reported microglial activation [237], cytokine production [231, 232, 237, 238], astrogliosis [223, 231, 239], altered neuronal morphology [229, 238, 239], synapse elimination [230], and neuronal loss [223, 230, 232] after SARS-CoV-2 infection. All of these processes are involved in AD. Even tau accumulation has been reported [240, 241], which is a hallmark of AD and other so called ‘tauopathies’.

Taken together, SARS-CoV-2 does display signs of neurodegenerative potential. However, it is possible that in vivo the neurodegenerative potential of SARS-CoV-2 is mediated by peripheral cytokine release and brain barrier disturbance (BBB, blood-CSF barrier) and cerebral hypoperfusion rather than by direct infection [236]. According to Matschke et. al, the severity of the histopathological changes they observed was not correlated with the presence of viral particles in the CNS which suggests that the peripheral inflammatory process is enough to cause CNS pathology [219]. Similarly, Käufer et al. have reported that intranasal SARS-CoV-2 infection caused microgliosis, tau hyperphosphorylation, and α-synuclein pathology in the hamster cortex even without CNS infection. Notably, the pathology persisted beyond virus clearance which could have implications for long-COVID and neurodegeneration [223].

#### Spirochete bacteria

Spirochetes are a group of spiral-shaped bacteria from the genera *Spirochaeta*, *Treponema*, *Borrelia* and *Leptospira*. Many members of this group are human pathogens that cause diseases such as leptospirosis, Lyme disease, relapsing fever, syphilis, and periodontitis. Cases for and against the involvement of spirochetes in neurodegenerative diseases have been raised.

For example, Miklossy et al. have suggested the involvement of spirochetes in AD [242–244]. They have reported that spirochetes, such as periodontal *Treponema* sp. and *Borrelia burgdorferi,* co-localize with Aβ and neurofibrillary tangles in AD brains, form plaque-like colonies, establish latent infections, disturb cerebral blood flow, and induce AD-related lesions and neuroinflammation [243, 244].

In vitro experiments by the same group on rat primary neurons, astrocytes, and microglia have shown that 2–8-week exposure to *Borrelia burgdorferi* induces AD-like changes, such as overexpression of the amyloid precursor protein (APP), tau hyperphosphorylation, and the accumulation of Aβ inclusions reminiscent of amyloid plaques. Interestingly, the presence of microglia increased Aβ accumulation, which highlights the importance of microglia in the neurodegeneration induced by spirochetes [242].

In contrast, Gutacker et al. found no evidence of *Borrelia burgdorferi* in the brains of ten AD patients [245]. Similarly, Forrester et al. found no geographic correlation between the incident rate of Lyme disease and death rate of neurodegenerative diseases in the United States [246]. However, neurodegenerative diseases are considered highly multifactorial, which makes such broad correlations difficult to assess. If it turns out multiple pathogen groups can trigger neurodegenerative changes in susceptible individuals, important correlations can get overlooked by studies focusing on one type of infection.

Syphilis is another pathogen that may be relevant to only a subset of patients. Syphilis is a sexually transmitted disease caused by the spirochete *Treponema pallidum*, which swept through Europe in the 1500s. It has since become rare due to effective antibiotic treatments. CNS presentation is relatively common at the early stages of syphilis [247]. It is characterized by sensorimotor symptoms such as abnormal gait, tremors, and numbness of the lower limbs accompanied by cognitive and mood dysfunction such as confusion, poor concentration, depression, and irritability. Interestingly, several cases of dementia caused by late neurosyphilis have been reported [248–259]. Commonly the patients are around 40–60-year-old at the time of diagnosis (early-onset for dementia) and they display a variety of neurological symptoms such as rapid cognitive decline, behavioral changes, and psychosis. In some patients, the symptoms ameliorate after treatment with antibiotics [248–259]. Thus, syphilis should be considered as a cause for dementia-like symptoms in infected individuals. Interestingly, asymptomatic neurosyphilis patients display different levels of Aβ42 and tau in the CSF compared to AD patients, which could be used as a biomarker to differentiate between the diseases as well as to determine the stage of neurosyphilis in a patient [260].

A few cases of suspected syphilis-induced ALS or ALS-mimic syndrome have also been reported [261–263]. However, determining whether these syndromes truly fill the criteria for ALS, and whether syphilis is causative in these cases, is beyond the expertise of the authors of this article [264]. Furthermore, anecdotal evidence suggests that other spirochete bacteria such as *Borrelia burgdorferi* can cause ALS-like pathology [265]. However, in general, Lyme disease does not seem to be associated with ALS [266, 267].

As for MS, one study has reported spirochete bacteria in the brains of four MS patients [268], but other evidence of spirochete involvement in MS is currently lacking.

#### Bacterial pneumonia

A link has been suggested between bacterial pneumonia and dementia. For example, a Taiwanese cohort study has reported a positive association between bacterial pneumonia and subsequent dementia risk (HR 2.83, 95% CI: 2.53–3.18), particularly between *Staphylococcus* pneumonia and vascular dementia (HR 5.4) and *Hemophilus* pneumonia and AD (HR 3.85, 95% CI: 1.66–8.96) [269]. Tate et al. have reported that hospitalization for pneumonia increased the risk of later developing dementia (aHR 1.9, 95% CI: 1.4–2.8, P < 0.0001) [270]. Neither study assessed whether the infection affected the CNS directly.

*Chlamydia pneumoniae* is a bacterial pathogen which causes respiratory infections, including pneumonia. It has been shown to sometimes enter the brain. Interestingly, Gérard et al. have reported *C. pneumoniae* in the neurons and glia of around 80–90% of late-onset AD patients and only 5–10% of healthy controls. Notably, the bacteria were detected in proximity to AD-related lesions such as amyloid plaques [164, 271, 272]. The authors did not list the cause of death for these patients. Since AD and dementia patients have a higher risk of hospitalization and mortality due to pneumonia than unaffected individuals [273, 274], more information would be needed to rule out the possibility that *C. pneumoniae* was present in the brains of these patients due to an infection secondary to AD.

However, Chacko et al. have shown that *C. pneumoniae* can infect the glia in olfactory and trigeminal nerves, olfactory bulb, and other brain areas of mice within 72 h of inoculation. They observed Aβ accumulation adjacent to bacteria in the olfactory system, and changes in neurodegeneration-relevant pathways [275]. Lopatko Lindman et al. have studied whether the Alzheimer’s disease risk allele APOE ε4 mediates the risk between *C. pneumoniae infection* and AD*.* They tested the presence of* C. pneumoniae* in the plasma of 360 AD and 360 matched controls from samples that had been collected on average 9.6 years before the diagnosis. They reported no association between *Chlamydia pneumoniae*, APOE ε4 allele, and the risk of AD [125]. In contrast, Gérard et al. have reported that *C. pneumoniae* burden is higher in the brains of AD patients that carry APOE ε4 allele compared to non-carriers [164]. Others have also reported that full length human APOE and derived peptides have direct antimicrobial function against Gram-negative bacteria [276, 277].

*C. pneumoniae* has also been studied in relation to many diseases with immunological etiology, including MS. However, reports have been published both for and against an association with MS, and clear conclusions cannot be drawn yet [278].

Finally, a study by Turkel et al. assessed the presence of *Chlamydia pneumoniae* in the serum of 51 PD patients and 37 matched controls. They found no statistically significant correlation between *C. pneumoniae* and PD [279].

#### Fungi

Fungi have received less attention in the study of neurodegenerative diseases than viruses and bacteria due to their lower relative abundance in the human microbiome. However, they are relevant human pathogens, particularly in immunocompromised individuals.

Most of the exploratory work on the involvement of fungi in neurodegenerative diseases has been done by a single research group lead by Luis Carrasco. They have detected multiple species of fungi in the brains of AD, PD, ALS, and MS patients.

For example, they detected *Candida albicans, Candida ortholopsis*, *Candida tropicalis*, *Cladosporium*, *Malassezia globosa*, *Malassezia restricta*, *Neosartorya hiratsukae*, *Phoma*, *Saccharomyces cerevisae*, and *Sclerotinia boreali*s in the brains of altogether ten AD patients but not in the same number of controls. Not all species were detected in all patients [280]. Similarly, they found antibodies against multiple species of fungi, as well as fungal proteins, and fungal 1,3-β-glucan in the serum of AD patients. Such signs of fungal infection were also present in healthy controls but at lower frequency [281]. Consistently, Yashkin et al. have reported that fungal infections increased the risk of AD (HR 1.98, 95% CI:1.89–1.92) in a dataset collected from a 5% sample of U.S. Medicare beneficiaries aged 65 + years [282] (the number of Medicare beneficiaries was over 65 million in 2023 [283]).

The group of Luis Carrasco have also reported the presence of *Botrytis, Candida, Fusarium and Malassezia* species in the brains of all six PD patients using nested PCR analysis. When they compared these results to a previously published control group (n = 12) [281], the level of fungal infection was higher in PD patients. They also detected chitin immunopositivity in samples from PD patients but not from controls [284]. Interestingly, they detected bacteria in the spinal cord, medulla, and motor cortex of the same PD patients, which suggests the possibility of polymicrobial infection in PD. While bacteria were also present in healthy controls (n = 9) the results clustered separately in principal component analysis which suggests that the infection profile was different in patients and controls [284].

The same group has also reported fungal intracellular structures such as yeasts and hyphae in the motor cortex, medulla, and spinal cord of all eleven assayed ALS patients. PCR and next generation sequencing revealed fungi from multiple genera: *Candida*, *Malassezia*, *Fusarium*, *Botrytis*, *Trichoderma*, and *Cryptococcus* [285]. Bacteria were present in the spinal cord, medulla, and motor cortex of the ALS patients but not in healthy controls, which suggests the possibility of polymicrobial infection also in ALS patients [286].

Similarly, polymicrobial infections of fungi and bacteria were present in the post mortem CNS tissues of all ten MS patients but not in controls. Particularly, the fungi *Trichosporon mucoides* was found in most MS patients [287]. Furthermore, the group has detected fungal RNA, antigens, and antibodies against multiple *Candida* species [288, 289] in the CSF of MS patients [288]. They also detected fungal DNA and β-1,3 glucan in patient blood and serum samples, respectively [289]. The same group reported that antigens for *Candida* increased the risk of MS 3.0 – 7.3-fold (depending on *Candida* species) in a cohort of 80 MS patients and 240 matched controls [290].

As for the mechanisms of fungi-induced neurodegeneration, *C. albicans* has been shown to cause AD-like pathology in cell culture models, including Aβ oligomerization and accumulation [291]. Furthermore, a study by Wu et al. showed that intravenous infection of *C. albicans* in mice causes mild memory impairment which resolves after fungal clearance. The impairment was associated with the production of proinflammatory cytokines (IL-1β, IL-6, TNF-α) as well as localized accumulation of soluble Aβ peptides, activated microglia, and astrocytes around the yeast cells [291] which suggests that neuroinflammation is a key factor in the observed pathology.

Interestingly, a couple studies have shown Aβ to be protective against *Candida* infections. Even though the suggested mechanisms differed between studies (reviewed in more detail in later section), the main finding was the same: Aβ peptides can inhibit the growth of *C. albicans *in vitro [291, 292]. Furthermore, full length APOE and derived peptides may also have a direct antimicrobial function against pathogens such as *Candida* yeasts [276, 277]. In fact, Vonk et al. have reported that APOE knock-out mice display higher mortality following *C. albicans* infection than their APOE wildtype controls [293]. Together this suggests a possible interplay between fungal infections and AD risk factors.

The involvement of fungal toxins in neurodegeneration has also been suggested. Fungal toxins such as gliotoxin and fumonisin have been shown to destroy glia such as astrocytes and oligodendrocytes, which could potentially lead to myelin loss in MS [294]. Gliotoxin from *Aspergillus fumigatus* has been reported to induce neuroinflammation and to aggravate clinical symptoms in experimental autoimmune encephalomyelitis mice (the most common mouse model for MS) [295]. It has also been reported to penetrate hiPSC model of the BBB and to impair the barrier function as measured by transendothelial electrical resistance (TEER) and fluorescein permeability [296].

Together, these data show that fungal pathogens are worth considering when searching for causes for neurodegenerative diseases. However, more research on the topic is needed in the future to better understand fungal CNS infections in seemingly immunocompetent individuals. Furthermore, the important exploratory work done by Carrasco et al. should be replicated by others to confirm and further elucidate the role of polymicrobial infections and different fungal species in neurodegeneration.

#### Toxoplasma gondii

*Toxoplasma gondii* is an obligate intracellular protozoan parasite, which is primarily hosted by cats and other small mammals. It infects around one third of the global human population [65, 66]. It forms chronic, cystic infection inside neurons, astrocytes and microglia of the brain. Normally, the host immune response keeps the parasite in latent state, and clinical manifestations (e.g. encephalitis) are typically seen only in immunocompromised patients.

*T. gondii* has been implicated in various diseases from psychiatric and mood disorders to epilepsy and autoimmune disorders*.* Reports regarding its involvement in neurodegenerative diseases have been conflicting [297]. For example, a Taiwanese cohort study of 200 dementia patients and 400 controls has reported a positive association between toxoplasmosis and dementia (HR 2.878, 95% CI = 1.71–4.97, *P* < 0.001). Furthermore, the treatment of toxoplasmosis by sulfadiazine or clindamycin was associated with a reduction in dementia risk [298]. A meta-analysis of eight studies (3239 subjects) suggests that *T. gondii* may be involved in AD (OR = 1.53, 95% CI: 1.07–2.18). However, the authors were worried about publication bias and called for more research on the subject [299]. In contrast, Wennberg et al. detected no association between *T. gondii* seropositivity and cognitive performance in 575 adults aged 41–97 years [121].

*T. gondii* infection has been reported to induce production of proinflammatory cytokines (TNF-α, IL-1β, IL-6), glial nodules, and hyperalgesia in wild type mice [300, 301]. Interestingly, studies of *T. gondii* infection in AD model mice have reported beneficial effects such as higher levels of anti-inflammatory cytokines, reduced Aβ tissue deposition, increased Aβ phagocytosis and degradation, reduced nitrite production from primary cultured microglial cells, and better performance in memory tests [302, 303].

Multiple studies have suggested that *T. gondii* infections might be associated with reduced risk of MS [304–307]. Due to the autoimmune nature of MS, the ‘hygiene hypothesis’ has been suggested as an explanation for reduced disease risk in *Toxoplasma* carriers. The idea is that parasites promote immunotolerance in the host by suppressing the innate and adaptive immune responses against themselves. The IFN-γ driven immune response is the main host response to *T. gondii* infection. However, the parasite can modulate transcription factors that regulate this pathway, e.g. signal transducer and activator of transcription (STAT) 1, 3, and 6. It also inhibits multiple apoptosis pathways, ROS production by nicotinamide adenine dinucleotide phosphate (NADPH) oxidases, as well as the nuclear factor kappa B (NF-κΒ) pathway, which regulates the production of many pro-inflammatory cytokines [308]. Activation of these same pathways have been implicated in the development of MS, however, their roles at different stages of the disease remain under debate [309, 310].

The hygiene hypothesis of MS has been criticized because of the link to EBV infections. [158]. However, different pathogens may have a different effect on the immune system. In fact, protozoan parasites have been explored as potential therapies for autoimmune diseases and allergies [311, 312] while the data on herpesviruses points mostly towards increased risk of autoimmunity [313]. In any case, conflicting reports on the involvement of *T. gondii* in MS have been published [314–317] and thus, the question remains under debate.

### Extra-CNS infections

Patients suffering from neurodegenerative diseases frequently display non-CNS infections such as gastric *H. pylori* infections [318–321], periodontal disease [322, 323], and changes in normal gut microbiome (dysbiosis) [324–334]. These changes have been linked to neurodegenerative diseases even in the absence of direct CNS infection [165, 335–338], and they share common mechanisms through which they can be detrimental to the CNS.

One key mechanism is the ability of these microbes to manipulate the extracellular matrix and the tight junctions of the host tissues. For example, gut microbes modulate the tight junctions of the gut epithelium by a variety of molecules. To illustrate, beneficial short-chain fatty acids (SCFAs), such as acetate, propionate and butyrate, are produced by fiber-digesting bacteria and they are utilized by host epithelial cells as an energy source. As a result, these bacteria facilitate normal epithelial barrier function [339, 340]. Furthermore, SCFAs are also known to cross the BBB where they modulate microglial function via G protein-coupled receptors [339]. Patients of neurodegenerative diseases often display reduction in the abundance of beneficial SCFA-producing bacteria [341, 342]. In turn, harmful gut microbes produce proinflammatory molecules such as LPS, cytokines, and prostaglandins, which disrupt the tightness of the epithelial barriers and can lead to a ‘leaky gut’. These same molecules can transfer to blood circulation and disrupt the BBB or cross into the CNS where they induce neuroinflammation [18–20].

Similarly, periodontal pathogens can produce molecules that disrupt BBB function [343]. For example, the Gram-negative bacteria *Porphyromonas gingivalis* and the spirochete *Treponema denticola* secrete molecules such as gingipains, toxic fimbriae, and dentilisin which allows them to modulate host tight junctions and to form a suitable habitat in the periodontal pocket [74, 344]. Interestingly, *P. gingivalis* has been associated with neurodegenerative diseases such as AD, PD and MS [165, 336–338, 345–351].

*P. gingivalis* has been detected in the post mortem brain tissue of AD patients. It has been reported to produce outer membrane vesicles carrying gingipains, LPS and toxic fimbriae, which can disrupt the BBB [345, 346]. Lei et al. reported that *P. gingivalis* infection and secreted gingipains increase transcytosis across the BBB by altering the expression of calveolin-1 and major facilitator superfamily domain containing 2a (Mfsd2a). They did not detect changes in occludin levels [344]. In contrast, Nonaka et al. have reported that *P. gingivalis*/gingipain disrupt tight junctions by direct degradation of zonula occludens-1 and occludin [344].

Once in the brain, gingipains cause AD-related changes such as as increase in Aβ and tau accumulation, modulate APP and tau processing, cause microglial activation and proliferation, induce IL-1β, TNF-α and IL-6 secretion, cleave APOE proteins, and induce neuronal degeneration and synapse loss [336, 346–348, 352]. *P. gingivalis*-derived LPS and inactivated bacteria also cause the secretion of nitric oxide and prostaglandin E2 from rat primary glial cells (95% astrocytes) [337]. Strikingly, peripheral administration of *P. gingivalis* is enough to cause a variety of neurodegenerative changes, which supports the involvement of indirect mechanisms such as outer membrane vesicles [165, 336–338]. For example, subcutaneous injection of *P. gingivalis* aggravated experimental autoimmune encephalomyelitis in MS model mice [337]. Oral *P. gingivalis* administration also led to microglial activation and loss of dopaminergic neurons in mice carrying the PD risk mutation LRRK2 R1441G. The mice displayed decreased expression of the tight junction protein zonula occludens-1 and increased expression of proinflammatory factors TNF-α, IL-1β, and α-synuclein in the colon. Increased serum IL-17A and increased brain IL-17A receptor expression implicates T helper (Th) 17 lymphocytes in the observed brain pathology [353]. Finally, *P. gingivalis* is not the only bacteria to produce harmful extracellular vesicles. Similar function has been attributed to the periodontal bacteria *Aggregatibacter actinomycetemcomitans* and *H. pylori* [335, 354–356].

Together, these results show that non-CNS infections can be as important for neurodegenerative diseases as CNS infections due to their potential to induce neuroinflammation. Thus, the search for causes for these diseases should not be limited to CNS infections, and non-CNS infections should also be considered when designing future treatments.

### Neurodegeneration-related aggregating proteins display antimicrobial properties

Many neurodegenerative diseases display characteristic protein aggregation in the nervous tissue. For example, AD is hallmarked by the accumulation of Aβ plaques and neurofibrillary tangles formed by hyperphosphorylated tau. Similarly, PD is hallmarked by α-synuclein inclusions, ALS by TDP-43 and FUS aggregation, and frontotemporal dementia with tau, TDP-43 and FUS aggregates. TDP-43 aggregates are also reported in some cases of AD. The role of these protein aggregates is still under debate, but traditionally they have been thought to be toxic. However, in recent years many of these aggregating amyloid proteins have been reported to have antimicrobial effects which suggests a role in the innate immune response.

The role of Aβ as an antimicrobial peptide was first suggested by Soscia et al. in 2010 [292]. They showed that synthetic and rodent amyloid-β peptides are effective against Gram -negative and -positive bacteria, and *C. albicans*. According to them, Aβ42 peptides showed greater efficiency against microbes than the less amyloidogenic Aβ40 peptides. They also showed that Aβ is able to bind anionic bacterial membranes, and that temporal lobe homogenates from AD patients inhibited the growth of *C. albicans* more than controls. Homogenates from the cerebellum of AD patients did not have the same effect, which makes sense considering that the cerebellum is not commonly affected by AD. Finally, the dose-dependent reduction on *C. albicans* growth by the temporal lobe homogenates from AD patients was abolished by pre-incubation with antibodies against Aβ [292].

Since then, Eimer et al. have reported that the heparin-binding site on soluble Aβ oligomers bind herpesvirus glycoproteins and leads to Aβ deposition and viral entrapment [357]. Bourgade et al. have reported that Aβ40 and Aβ42 peptides inhibit the replication of HSV-1, an enveloped virus, but not that of non-enveloped human adenovirus [358]. Their results suggest that Aβ may exert its antimicrobial function through the pathogen surface membranes, and it may only be effective against certain pathogens.

The effectivity of Aβ against HSV-1 has been called into question by Bocharova et al. who reported that Aβ failed to protect AD model mice (5xFAD) from HSV-1 infection. These mice carry mutations in APP which leads to Aβ overexpression and early-onset AD in humans. Bocharova et al. did not observe any colocalization of Aβ aggregates and viral particles, or significant differences in survival rate between infected 5xFAD and wildtype mice. However, they did report that HSV-1 invasion was limited in areas of high Aβ burden which they attributed to increased phagocytosis by microglia chronically activated by Aβ [359].

This result is in line with Wu et al. who observed that synthetic and human Aβ40 and Aβ42 peptides inhibited the growth of *C. albicans* through an interaction with immortalized murine BV-2 microglia. Mainly, they observed that Aβ treatment increased the phagocytosis of yeast particles by BV-2 microglia and induced the secretion of presently unknown fungistatic compounds from the same cells [291]. Unlike Soscia et al. they did not observe a direct fungistatic effect of Aβ [291].

The study by Wu et al. suggests that Aβ facilitates the fast innate immune response. In short, they showed that a knockout of APP in mice impaired the clearance of *C. albicans* during the early days of the infection (days 4–7). The defect was associated with hypothermia and impaired secretion of proinflammatory cytokines. In contrast, *C. albicans* clearance was enhanced 5xFAD mice known for the overexpression of APP and Aβ. However, since all mice were able to clear the *C. albicans* infection by day 10, these data suggest that Aβ is not obligatory for fungal clearance but instead boosts the fast immune response [291]. Interestingly, Bocharova et.al. also observed better survival rate within the first 140 h (day 5) after HSV-1 infection in 7–10-month-old 5xFAD mice compared to wildtype littermates. However, the result was not statistically significant [359].

Taken together, these data support the role of Aβ as an innate immune alarm molecule that mediates acute response to microbial infection. Similar function has been suggested to other neurodegeneration-related aggregating proteins as we will see below.

The microtubule-associated protein Tau is involved in cytoskeletal organization. It has been linked to many neurodegenerative diseases termed ‘tauopathies’ due to its propensity to hyperphosphorylate and to form intracellular inclusions called ‘neurofibrillary tangles’. In AD, the accumulation of neurofibrillary tangles is thought to be secondary to Aβ pathology. Kobayashi et al. have reported that small synthetic peptides carrying microtubule binding sites of the tau 4R isoform display potent antimicrobial effects against *Staphylococcus aureus* and *Escherichia coli*. The effect was strengthened by the addition of tandem repeat sequences or sequences for nuclear localization or laminin receptor binding site. The addition of sequences for nuclear localization or laminin receptor binding site also made the peptides effective against *C. albicans* [360]. Similarly, Kanagasingam et al. have reported that some phosphorylated tau peptides, but not all, reduced the viability of the periodontal bacteria *P. gingivalis*. The effective peptides were more prone to form β-sheet structures than peptides that did not show antimicrobial effects [361].

Alam et al. report that α-synuclein, which is secreted from enteric nervous system neurons, is needed for normal peritoneal immune response. In their study, α-synuclein knockout mice displayed reduced immune cell invasion of peritoneal cavity and reduced production of proinflammatory cytokines compared to controls following injection with bacterial peptidoglycan. Furthermore, the exogenous administration of α-synuclein in the peritoneal cavity of the knockout mice promoted leukocyte recruitment which suggests that α-synuclein can act as an immune system alarm molecule (alarmin). In addition, α-synuclein aggregation has been found in the gut and it has been suggested that α-synuclein pathology could originate in the gut and travel to the brain via afferent neurons [362].

FUS and TDP-43 are RNA-binding proteins which are known to localize in stress granules [363, 364]: intracellular assemblies formed by RNA, ribosomes and RNA-binding proteins that repress protein translation during stress. Since viruses require the host cell machinery for replication, the repression of host protein synthesis can protect the system from the spread of the infection [365].

Interestingly, it has been reported that infection with the enterovirus coxsackievirus B3 induces the cleavage and translocation of both TDP-43 and FUS. Furthermore, the knockout of TDP-43 or FUS induces an increase in virus titer, suggesting these proteins can repress virus replication [366, 367].

The connection between FUS and the innate immune system is further illustrated by the fact that poly(I:C), stress granule-inducing respiratory syncytial virus, and type I interferon are all able to induce FUS assembly in in vitro cell lines [368]. FUS knockout causes a reduction in the production in type 1 interferon and other pro-inflammatory cytokines following similar infection-mimicking treatments [367].

Similarly, Dunker et al. have reported that TDP-43 is required for the regulation of the accumulation of immunostimulatory double-stranded RNA, and for the inhibition of interferon-mediated necrotic cell death [369]. Interestingly, a recent article by Licht-Murava et al. reports that aberrant TDP-43 accumulation in hippocampal astrocytes causes memory impairment by inducing interferon-mediated antiviral response [370]. It suggests that TDP-43 may be useful or harmful depending on the situation. Cabrera et al. have reported that latent HSV-2 infection in mouse spinal cord was not associated with changes in TDP-43 or FUS despite other signs of immune activation and ALS pathology such as leukocyte infiltration, microglial changes near motor neurons and reduction in C9orf72 levels [371]. Thus, a lot more research on the topic is needed.

### Methodologies and future experimental avenues

In the future, population level association studies are expected to move from associations between a single pathogen and a neurodegenerative disease (e.g. HSV-1 and AD) to more complex models that consider a person’s lifetime infection burden and polygenic risk score [372]. Also moving the focus from severe infections towards more prevalent milder infections will further clarify the interaction between infections and neurodegenerative diseases. Such studies would require large-scale screening of the healthy population so that elusive patterns between asymptomatic or mild infections with neurodegeneration could be uncovered. When done consistently over long periods of time, such datasets could also elucidate the temporal patterns of disease development. However, such projects are labor- and cost-intensive, especially when both sexes and ethnically different human populations are represented in studies. The most feasible way to carry out such studies is their execution as part of large operations such as biobanks.

Simultaneously, experimental approaches are needed to advance from mere associations towards more mechanistic insight. Progressing from broad claims such as ‘infections cause neuroinflammation, which leads to neurodegeneration’ to clearer insight on why some individuals are affected while others are not, and to more detailed processes that lead to distinct neurodegenerative diseases, requires more studies where animals or cell culture models carrying different risk factors are infected with different pathogens. Including polymicrobial infections [373] and their combinations with different genetic risk factors into cell and animal models are likely to increase our understanding of possible heterogeneous susceptibility to infection-associated neurodegeneration.

Both animal models and stem-cell based methods have their advantages. For example, hIPSC-derived 2D and 3D models allow us to study infections on human brain cells, to overcome challenges related to species differences, and to elucidate the roles of different human cells types, e.g. neurons, astrocytes and microglia [374]. Furthermore, stem cell lines can be engineered to carry relevant genetic mutations, or they can be directly collected from affected donors e.g. AD patients carrying APOE mutations [374]. The limitation of in vitro stem cell models is that the peripheral immune component and the microbiome are often missing. Stem cell models also do not offer any information on whether the cell or molecular level change will penetrate to the level of behavior, e.g. cognitive function or motor symptoms. Xenotransplantation of human cells into mice (chimeric models) could solve this problem [375]. However, the xenotransplantation of human cells is currently only possible in immunocompromised animals [375] which limits the relevance of these models when studying infections. Thus, traditional in vivo animal models are still needed. For example, animal models of latent and reactivating virus infections have a lot to give to the field [27, 28].

Considering the importance of BBB disturbance in neurodegenerative diseases, improved models and detection methods are needed to elucidate the role of infections in this complex process. In humans, the measurement of BBB integrity in vivo is still emerging. However, imaging techniques such as positron emission tomography (PET) or magnetic resonance imaging (MRI) in combination with small molecule contrast agents show potential [376]. In animal studies, a wider range of dyes, diffusible tagged molecules, and in vivo and ex vivo imaging techniques are available [377]. In cell culture, trans-epithelial electrical resistance (TEER) and immunocytochemistry of tight junction proteins are common ways to study barrier integrity [378]. Stem cell-derived BBB-on-a-chip models are also under development [379].

Finally, the addition of pharmacological interventions into animal and cell culture-based models can be very powerful. Combined use of antimicrobials and anti-inflammatory drugs, such as resveratrol [380–382], curcumin [383], or omega-3 fatty acids [384], should tell us more about the role of each component in neurodegeneration. However, some compounds, such as resveratrol [382] and some antifungals [385], display both antiviral and anti-inflammatory properties. Thus, any experimental procedures need to be carefully planned.

## Conclusions

The current literature shows that a broad range of pathogenic CNS and extra-CNS infections are linked to AD, PD, ALS, and MS. While some pathogens seem to be more strongly associated with certain diseases than others (e.g. EBV and MS), no direct causality can yet be assigned. Instead, the current data suggests that the neurodegenerative potential of CNS pathogenic infections is tied to the neuroinflammation which follows. The current understanding of the antimicrobial effects of neurodegeneration-associated aggregating proteins supports this view. Furthermore, many of us carry CNS infections without ever developing neurodegenerative disease, which suggests that a combination of genetic susceptibility factors and environmental triggers, such as infections, are needed for the development of neurodegenerative pathologies. Going forward, more research on the interplay between genetic risk factors and CNS infections is needed, as well as on the cumulative effect of multiple simultaneous or subsequent infections. While it is impossible to stop people from ever getting infections again, antimicrobial and anti-inflammatory drugs and dietary supplements have shown some promise in the treatment of neurodegenerative diseases. Thus, better understanding of the role of CNS infections in neurodegenerative diseases could help us detect vulnerable individuals and to target a combination of antimicrobial and anti-inflammatory therapies to those that could most benefit from them.

## Data Availability

Not applicable.

## Abbreviations

AD: Alzheimer’s disease
Aβ: Amyloid-beta
(a)HR: (Adjusted) hazard ratio
ALS: Amyotrophic lateral sclerosis
APP: Amyloid precursor protein
APOE: Apolipoprotein E
BBB: Blood–brain barrier
95% CI: 95% Confidence interval
CMV: Cytomegalovirus
CNS: Central nervous system
EBV: Epstein-Barr virus
FUS: Fused in sarcoma
HHV: Human herpesvirus
HAND: Hiv-associated neurocognitive disorder
HIV/AIDS: Human immunodeficiency virus/acquired immune deficiency syndrome
HPV: Human papillomavirus
HSV: Herpes simplex virus
iPSC: Induced pluripotent stem cell
LPS: Lipopolysaccharide
MBP: Myelin basic protein
MS: Multiple sclerosis
(a)OR: (Adjusted) odds ratio
PD: Parkinson’s disease
ROS: Reactive oxygen species
RT-qPCR: Real time quantitative polymerase chain reaction
SARS-CoV-2: Severe acute respiratory syndrome coronavirus 2
SCFA: Short-chain fatty acid
SOD-1: Superoxide dismutase 1
TDP-43: Transitive response DNA-binding protein 43
TREM2: Triggering receptor expressed on myeloid cells 2
VZV: Varicella zoster virus

## References

[1] Seaks CE, Wilcock DM (2020). Infectious hypothesis of Alzheimer disease. PLoS Pathog.

[2] Cabezudo D, Baekelandt V, Lobbestael E (2020). Multiple-hit hypothesis in Parkinson’s disease: LRRK2 and inflammation. Front Neurosci.

[3] Boyd RJ, Avramopoulos D, Jantzie LL, McCallion AS (2022). Neuroinflammation represents a common theme amongst genetic and environmental risk factors for Alzheimer and Parkinson diseases. J Neuroinflammation.

[4] McEntire CRS, Song KW, McInnis RP, Rhee JY, Young M, Williams E (2021). Neurologic manifestations of the World Health Organization’s list of pandemic and epidemic diseases. Front Neurol.

[5] Ceban F, Ling S, Lui LMW, Lee Y, Gill H, Teopiz KM (2022). Fatigue and cognitive impairment in Post-COVID-19 Syndrome: a systematic review and meta-analysis. Brain Behav Immun.

[6] Le Govic Y, Demey B, Cassereau J, Bahn YS, Papon N (2022). Pathogens infecting the central nervous system. PLoS Pathog.

[7] Dando SJ, Mackay-Sim A, Norton R, Currie BJ, St John JA, Ekberg JAK (2014). Pathogens penetrating the central nervous system: infection pathways and the cellular and molecular mechanisms of invasion. Clin Microbiol Rev.

[8] Levine KS, Leonard HL, Blauwendraat C, Iwaki H, Johnson N, Bandres-Ciga S (2023). Virus exposure and neurodegenerative disease risk across national biobanks. Neuron.

[9] Vora NM, Holman RC, Mehal JM, Steiner CA, Blanton J, Sejvar J (2014). Burden of encephalitis-associated hospitalizations in the United States, 1998–2010. Neurology.

[10] Rezaei SJ, Mateen FJ (2021). Encephalitis and meningitis in Western Africa: a scoping review of pathogens. Trop Med Int Health.

[11] Rocha ND, de Moura SK, da Silva GAB, Mattiello R, Sato DK (2023). Neurological sequelae after encephalitis associated with herpes simplex virus in children: systematic review and meta-analysis. BMC Infect Dis.

[12] Campbell G, Hills S, Fischer M, Jacobson J, Hoke C, Hombach J (2011). Estimated global incidence of Japanese encephalitis: a systematic review. Bull World Health Organ.

[13] Antonello RM, Riccardi N (2022). How we deal with Staphylococcus aureus (MSSA, MRSA) central nervous system infections. Front Biosci Scholar.

[14] Peterson CT (2020). Dysfunction of the microbiota-gut-brain axis in neurodegenerative disease: the promise of therapeutic modulation with prebiotics, medicinal herbs, probiotics, and synbiotics. J Evid Based Integr Med..

[15] Galea I (2021). The blood-brain barrier in systemic infection and inflammation. Cell Mol Immunol.

[16] Varatharaj A, Galea I (2017). The blood-brain barrier in systemic inflammation. Brain Behav Immun.

[17] Skelly DT, Hennessy E, Dansereau MA, Cunningham C (2013). A systematic analysis of the peripheral and CNS effects of systemic LPS, IL-1β, [corrected] TNF-α and IL-6 challenges in C57BL/6 mice. PLoS ONE.

[18] Sankowski R, Mader S, Valdés-Ferrer SI (2015). Systemic inflammation and the brain: novel roles of genetic, molecular, and environmental cues as drivers of neurodegeneration. Front Cell Neurosci.

[19] Wong ML, Rettori V, AL-Shekhlee A, Bongiorno PB, Canteros G, McCann SM (1996). Inducible nitric oxide synthase gene expression in the brain during systemic inflammation. Nat Med.

[20] Püntener U, Booth SG, Perry VH, Teeling JL (2012). Long-term impact of systemic bacterial infection on the cerebral vasculature and microglia. J Neuroinflammation.

[21] Li F, Wang Y, Yu L, Cao S, Wang K, Yuan J (2015). Viral infection of the central nervous system and neuroinflammation precede blood-brain barrier disruption during Japanese encephalitis virus infection. J Virol.

[22] Vale A do, Cabanes D, Sousa S. Bacterial toxins as pathogen weapons against phagocytes. Front Microbiol. 2016;7: 172814 10.3389/fmicb.2016.00042 PMC4734073 26870008

[23] Chen WA, Dou Y, Fletcher HM, Boskovic DS (2023). Local and Systemic effects of *Porphyromonas gingivalis* infection. Microorganisms.

[24] Cangui-Panchi SP, Ñacato-Toapanta AL, Enríquez-Martínez LJ, Salinas-Delgado GA, Reyes J, Garzon-Chavez D (2023). Battle royale: Immune response on biofilms—host-pathogen interactions. Curr Res Immunol.

[25] Egorov AI, Converse RR, Griffin SM, Styles JN, Sams E, Hudgens E (2021). Latent *Toxoplasma gondii* infections are associated with elevated biomarkers of inflammation and vascular injury. BMC Infect Dis.

[26] Hatano T, Sano D, Takahashi H, Oridate N (2021). Pathogenic role of immune evasion and integration of human papillomavirus in oropharyngeal cancer. Microorganisms.

[27] Protto V, Tramutola A, Fabiani M, Marcocci ME, Napoletani G, Iavarone F (2020). Multiple herpes simplex virus-1 (HSV-1) reactivations induce protein oxidative damage in mouse brain: novel mechanisms for Alzheimer’s disease progression. Microorganisms.

[28] De Chiara G, Piacentini R, Fabiani M, Mastrodonato A, Marcocci ME, Limongi D (2019). Recurrent herpes simplex virus-1 infection induces hallmarks of neurodegeneration and cognitive deficits in mice. PLoS Pathog.

[29] zur Hausen H (2009). Papillomaviruses in the causation of human cancers—a brief historical account. Virology.

[30] Mui U, Haley C, Tyring S (2017). Viral oncology: molecular biology and pathogenesis. J Clin Med.

[31] Chang AH, Parsonnet J (2010). Role of bacteria in oncogenesis. Clin Microbiol Rev.

[32] Mentzer AJ, Brenner N, Allen N, Littlejohns TJ, Chong AY, Cortes A (2022). Identification of host–pathogen-disease relationships using a scalable multiplex serology platform in UK Biobank. Nat Commun.

[33] Doi Y, Ninomiya T, Hata J, Yonemoto K, Tanizaki Y, Arima H (2009). Seroprevalence of herpes simplex virus 1 and 2 in a population-based cohort in Japan. J Epidemiol.

[34] Sukik L, Alyafei M, Harfouche M, Abu-Raddad LJ (2019). Herpes simplex virus type 1 epidemiology in Latin America and the Caribbean: systematic review and meta-analytics. PLoS ONE.

[35] Khadr L, Harfouche M, Omori R, Schwarzer G, Chemaitelly H, Abu-Raddad LJ (2019). The epidemiology of herpes simplex virus type 1 in Asia: systematic review, meta-analyses, and meta-regressions. Clin Infect Dis.

[36] Chaabane S, Harfouche M, Chemaitelly H, Schwarzer G, Abu-Raddad LJ (2019). Herpes simplex virus type 1 epidemiology in the Middle East and North Africa: systematic review, meta-analyses, and meta-regressions. Sci Rep.

[37] Harfouche M, Maalmi H, Abu-Raddad LJ (2021). Epidemiology of herpes simplex virus type 2 in Latin America and the Caribbean: systematic review, meta-analyses and metaregressions. Sex Transm Infect.

[38] James C, Harfouche M, Welton NJ, Turner KM, Abu-Raddad LJ, Gottlieb SL (2020). Herpes simplex virus: global infection prevalence and incidence estimates, 2016. Bull World Health Organ.

[39] Kang CI, Choi CM, Park TS, Lee DJ, Oh M don, Choe KW. Incidence of herpes zoster and seroprevalence of varicella-zoster virus in young adults of South Korea. Int J Infect Dis. 2008;12(3):245–7. 10.1016/j.ijid.2007.08.002 17950022

[40] Reynolds MA, Kruszon-Moran D, Jumaan A, Schmid DS, McQuillan GM (2010). Varicella seroprevalence in the U.S.: data from the National Health and Nutrition Examination Survey, 1999–2004. Public Health Rep.

[41] van Rijckevorsel GG, Damen M, Sonder GJ, van der Loeff MFS, van den Hoek A (2012). Seroprevalence of varicella-zoster virus and predictors for seronegativity in the Amsterdam adult population. BMC Infect Dis.

[42] Amjadi O, Rafiei A, Haghshenas M, Navaei RA, Valadan R, Hosseini-Khah Z (2017). A systematic review and meta-analysis of seroprevalence of varicella zoster virus: a nationwide population-based study. J Clin Virol.

[43] Irekeola AA, Wada Y, Mohamud R, Mat Lazim N, Yean CY (2022). Prevalence of EBV infection in 1157 diseased cohorts in Nigeria: a systematic review and meta-analysis. Indian J Med Microbiol.

[44] Lai PK, Mackay-Scollay EM, Alpers MP (1975). Epidemiological studies of Epstein-Barr herpesvirus infection in Western Australia. J Hygiene.

[45] Xiong G, Zhang B, Yun HM, Zhou H, Zhen CL, Sheng FQ (2014). Epstein-barr virus (EBV) Infection in Chinese children: a retrospective study of age-specific prevalence. PLoS ONE.

[46] Smatti MK, Yassine HM, AbuOdeh R, AlMarawani A, Taleb SA, Althani AA (2017). Prevalence and molecular profiling of Epstein Barr virus (EBV) among healthy blood donors from different nationalities in Qatar. PLoS ONE.

[47] Fowler K, Mucha J, Neumann M, Lewandowski W, Kaczanowska M, Grys M (2022). A systematic literature review of the global seroprevalence of cytomegalovirus: possible implications for treatment, screening, and vaccine development. BMC Public Health.

[48] Bates M, Brantsaeter AB (2016). Human cytomegalovirus (CMV) in Africa: a neglected but important pathogen. J Virus Erad.

[49] Braun DK, Dominguez G, Pellett PE (1997). Human herpesvirus 6. Clin Microbiol Rev.

[50] Okuno T, Takahashi K, Balachandra K, Shiraki K, Yamanishi K, Takahashi M (1989). Seroepidemiology of human herpesvirus 6 infection in normal children and adults. J Clin Microbiol.

[51] Al-Sadeq DW, Zedan HT, Aldewik N, Elkhider A, Hicazi A, Younes N (2022). Human herpes simplex virus-6 (HHV-6) detection and seroprevalence among Qatari nationals and immigrants residing in Qatar. IJID Regions.

[52] Kuusisto H, Hyöty H, Kares S, Kinnunen E, Elovaara I (2008). Human herpes virus 6 and multiple sclerosis: a Finnish twin study. Mult Scler J.

[53] de Freitas RB, Linhares AC (1997). Prevalence of human herpesvirus 6 antibody in the population of Belém, Pará, northern Brazil. Trans R Soc Trop Med Hyg.

[54] Ablashi DV, Berneman ZN, Kramarsky B, Whitman J, Asano Y, Pearson GR (1995). Human herpesvirus-7 (HHV-7): current status. Clin Diagn Virol.

[55] Krueger GRF, Koch B, Leyssens N, Berneman Z, Rojo J, Horwitz C (1998). Comparison of Seroprevalences of Human Herpesvirus-6 and -7 in Healthy Blood Donors from Nine Countries. Vox Sang.

[56] Dollard SC, Butler LM, Jones AMG, Mermin JH, Chidzonga M, Chipato T (2010). Substantial regional differences in human herpesvirus 8 seroprevalence in sub-Saharan Africa: insights on the origin of the “Kaposi’s sarcoma belt”. Int J Cancer.

[57] Pellett PE, Wright DJ, Engels EA, Ablashi DV, Dollard SC, Forghani B (2003). Multicenter comparison of serologic assays and estimation of human herpesvirus 8 seroprevalence among US blood donors. Transfusion (Paris).

[58] Gambús G, Bourboulia D, Esteve A, Lahoz R, Rodriguez C, Bolao F (2001). Prevalence and distribution of HHV-8 in different subpopulations, with and without HIV infection. Spain AIDS.

[59] Chavoshpour-Mamaghani S, Shoja Z, Mollaei-Kandelous Y, Sharifian K, Jalilvand S (2021). The prevalence of human herpesvirus 8 in normal, premalignant, and malignant cervical samples of Iranian women. Virol J.

[60] Ablashi D, Chatlynne L, Cooper H, Thomas D, Yadav M, Norhanom AW (1999). Seroprevalence of human herpesvirus-8 (HHV-8) in countries of Southeast Asia compared to the USA, the Caribbean and Africa. Br J Cancer.

[61] de Sanjosé S, Diaz M, Castellsagué X, Clifford G, Bruni L, Muñoz N (2007). Worldwide prevalence and genotype distribution of cervical human papillomavirus DNA in women with normal cytology: a meta-analysis. Lancet Infect Dis.

[62] Moreira ED, Giuliano AR, Palefsky J, Flores CA, Goldstone S, Ferris D (2014). Incidence, clearance, and disease progression of genital human papillomavirus infection in heterosexual men. J Infect Dis.

[63] Bruni L, Albero G, Rowley J, Alemany L, Arbyn M, Giuliano AR (2023). Global and regional estimates of genital human papillomavirus prevalence among men: a systematic review and meta-analysis. Lancet Glob Health.

[64] Kombe Kombe AJ, Li B, Zahid A, Mengist HM, Bounda GA, Zhou Y (2021). Epidemiology and Burden of Human Papillomavirus and Related Diseases, Molecular Pathogenesis, and Vaccine Evaluation. Front Public Health.

[65] Pappas G, Roussos N, Falagas ME (2009). Toxoplasmosis snapshots: global status of *Toxoplasma gondii* seroprevalence and implications for pregnancy and congenital toxoplasmosis. Int J Parasitol.

[66] Siponen A, Kinnunen PM, Koort J, Kallio-Kokko H, Vapalahti O, Virtala A (2019). *Toxoplasma gondii* seroprevalence in veterinarians in Finland: Older age, living in the countryside, tasting beef during cooking and not doing small animal practice associated with seropositivity. Zoonoses Public Health.

[67] Bigna JJ, Tochie JN, Tounouga DN, Bekolo AO, Ymele NS, Youda EL (2020). Global, regional, and country seroprevalence of *Toxoplasma gondii* in pregnant women: a systematic review, modelling and meta-analysis. Sci Rep.

[68] Stopić M, Štajner T, Marković-Denić L, Nikolić V, Djilas I, Srzentić SJ, et al. Epidemiology of toxoplasmosis in SERBIA: a cross-sectional study on blood donors. microorganisms. 2022;10(3):492 10.3390/microorganisms10030492 PMC8948843 35336068

[69] Karshima SN, Karshima MN (2020). Human Toxoplasma gondii infection in Nigeria: a systematic review and meta-analysis of data published between 1960 and 2019. BMC Public Health.

[70] Ghazaei C (2022). Advances in the study of bacterial toxins, their roles and mechanisms in pathogenesis. Malays J Med Sci.

[71] Žerovnik E (2021). Viroporins vs. other pore-forming proteins: what lessons can we take?. Front Chem.

[72] Dong D, Xie W, Liu M (2020). Alteration of cell junctions during viral infection. Thorac Cancer.

[73] Cossart P, Helenius A (2014). Endocytosis of viruses and bacteria. Cold Spring Harb Perspect Biol.

[74] Chi B, Qi M, Kuramitsu HK (2003). Role of dentilisin in *Treponema denticola* epithelial cell layer penetration. Res Microbiol.

[75] Salimi H, Klein RS, Mitoma H, Manto M (2019). Disruption of the blood-brain barrier during neuroinflammatory and neuroinfectious diseases. Neuroimmune diseases: from cells to the living brain.

[76] Tang J, Frascaroli G, Zhou X, Knickmann J, Brune W (2021). Cell fusion and syncytium formation in betaherpesvirus infection. Viruses.

[77] Shieh JTC, Martín J, Baltuch G, Malim MH, González-Scarano F (2000). Determinants of syncytium formation in microglia by human immunodeficiency virus type 1: role of the V1/V2 domains. J Virol.

[78] Buchrieser J, Dufloo J, Hubert M, Monel B, Planas D, Rajah MM (2020). Syncytia formation by SARS-CoV-2-infected cells. EMBO J.

[79] Walsh D, Naghavi MH (2019). Exploitation of cytoskeletal networks during early viral infection. Trends Microbiol.

[80] Richards A, Berth SH, Brady S, Morfini G (2021). Engagement of neurotropic viruses in fast axonal transport: mechanisms, potential role of host kinases and implications for neuronal dysfunction. Front Cell Neurosci.

[81] Rozman B, Fisher T, Stern-Ginossar N (2023). Translation—a tug of war during viral infection. Mol Cell.

[82] Sonninen TM, Goldsteins G, Laham-Karam N, Koistinaho J, Lehtonen Š (2020). Proteostasis disturbances and inflammation in neurodegenerative diseases. Cells.

[83] Cohen JI (2020). Herpesvirus latency. J Clin Investig.

[84] Doty KR, Guillot-Sestier MV, Town T (2015). The role of the immune system in neurodegenerative disorders: adaptive or maladaptive?. Brain Res.

[85] https://www.who.int/news-room/fact-sheets/detail/dementia. 2024.

[86] Farrer LA, Cupples LA, Haines JL, Hyman B, Kukull WA, Mayeux R, et al. Effects of age, sex, and ethnicity on the association between apolipoprotein E genotype and Alzheimer disease A meta-analysis. APOE and Alzheimer Disease Meta Analysis Consortium. JAMA. 278(16):1349–56. 9343467

[87] Yen JHJ, Yu ICI (2023). The role of ApoE-mediated microglial lipid metabolism in brain aging and disease. Immunometabolism.

[88] Parhizkar S, Holtzman DM (2022). APOE mediated neuroinflammation and neurodegeneration in Alzheimer’s disease. Semin Immunol.

[89] Nott A, Holtman IR (2023). Genetic insights into immune mechanisms of Alzheimer’s and Parkinson’s disease. Front Immunol.

[90] Hansen DV, Hanson JE, Sheng M (2018). Microglia in Alzheimer’s disease. J Cell Biol.

[91] Efthymiou AG, Goate AM (2017). Late onset Alzheimer’s disease genetics implicates microglial pathways in disease risk. Mol Neurodegener.

[92] Jin SC, Benitez BA, Karch CM, Cooper B, Skorupa T, Carrell D (2014). Coding variants in TREM2 increase risk for Alzheimer’s disease. Hum Mol Genet.

[93] Li R, Wang X, He P (2021). The most prevalent rare coding variants of TREM2 conferring risk of Alzheimer’s disease: a systematic review and meta-analysis. Exp Ther Med.

[94] https://www.who.int/news-room/fact-sheets/detail/parkinson-disease. 2024.

[95] Billingsley KJ, Bandres-Ciga S, Saez-Atienzar S, Singleton AB (2018). Genetic risk factors in Parkinson’s disease. Cell Tissue Res.

[96] Shutinoski B, Hakimi M, Harmsen IE, Lunn M, Rocha J, Lengacher N (2019). Lrrk2 alleles modulate inflammation during microbial infection of mice in a sex-dependent manner. Sci Transl Med..

[97] Ahmadi Rastegar D, Hughes LP, Perera G, Keshiya S, Zhong S, Gao J (2022). Effect of LRRK2 protein and activity on stimulated cytokines in human monocytes and macrophages. NPJ Parkinsons Dis.

[98] Umoh ME, Fournier C, Li Y, Polak M, Shaw L, Landers JE (2016). Comparative analysis of C9orf72 and sporadic disease in an ALS clinic population. Neurology.

[99] Smeyers J, Banchi EG, Latouche M (2021). C9ORF72: what it is, what it does, and why it matters. Front Cell Neurosci.

[100] Balendra R, Isaacs AM (2018). C9orf72-mediated ALS and FTD: multiple pathways to disease. Nat Rev Neurol.

[101] Javad TM, Vodjgani M, Salehi Z, Izad M (2020). The influence of reactive oxygen species in the immune system and pathogenesis of multiple sclerosis. Autoimmune Dis..

[102] Bhattacharya A, Hegazy AN, Deigendesch N, Kosack L, Cupovic J, Kandasamy RK (2015). Superoxide dismutase 1 protects hepatocytes from type i interferon-driven oxidative damage. Immunity.

[103] Walton C, King R, Rechtman L, Kaye W, Leray E, Marrie RA (2020). Rising prevalence of multiple sclerosis worldwide: insights from the Atlas of MS, third edition. Multiple Sclerosis J.

[104] Parnell GP, Booth DR (2017). The multiple sclerosis (MS) genetic risk factors indicate both acquired and innate immune cell subsets contribute to MS pathogenesis and identify novel therapeutic opportunities. Front Immunol.

[105] Soldan SS, Lieberman PM (2023). Epstein-Barr virus and multiple sclerosis. Nat Rev Microbiol.

[106] Lanz TV, Brewer RC, Ho PP, Moon JS, Jude KM, Fernandez D (2022). Clonally expanded B cells in multiple sclerosis bind EBV EBNA1 and GlialCAM. Nature.

[107] Koton S, Pike JR, Johansen M, Knopman DS, Lakshminarayan K, Mosley T (2022). Association of ischemic stroke incidence, severity, and recurrence with dementia in the atherosclerosis risk in communities cohort study. JAMA Neurol.

[108] Kuźma E, Lourida I, Moore SF, Levine DA, Ukoumunne OC, Llewellyn DJ (2018). Stroke and dementia risk: a systematic review and meta-analysis. Alzheimer’s Dementia.

[109] Brain J, Greene L, Tang EYH, Louise J, Salter A, Beach S (2023). Cardiovascular disease, associated risk factors, and risk of dementia: an umbrella review of meta-analyses. Front Epidemiol.

[110] Hung CM, Li YC, Chen HJ, Lu K, Liang CL, Liliang PC (2018). Risk of dementia in patients with primary insomnia: a nationwide population-based case-control study. BMC Psychiatry.

[111] Tan X, Åkerstedt T, Lagerros YT, Åkerstedt AM, Bellocco R, Adami HO (2023). Interactive association between insomnia symptoms and sleep duration for the risk of dementia—a prospective study in the Swedish National March Cohort. Age Ageing.

[112] Sabia S, Fayosse A, Dumurgier J, van Hees VT, Paquet C, Sommerlad A (2021). Association of sleep duration in middle and old age with incidence of dementia. Nat Commun.

[113] Murphy MJ, Fani L, Ikram MK, Ghanbari M, Ikram MA (2021). Herpes simplex virus 1 and the risk of dementia: a population-based study. Sci Rep.

[114] Schmidt SAJ, Veres K, Sørensen HT, Obel N, Henderson VW (2022). Incident herpes zoster and risk of dementia: a population-based Danish cohort study. Neurology.

[115] Shim Y, Park M, Kim J (2022). Increased incidence of dementia following herpesvirus infection in the Korean population. Medicine.

[116] Sipilä PN, Heikkilä N, Lindbohm JV, Hakulinen C, Vahtera J, Elovainio M (2021). Hospital-treated infectious diseases and the risk of dementia: a large, multicohort, observational study with a replication cohort. Lancet Infect Dis.

[117] Bae S, Yun SC, Kim MC, Yoon W, Lim JS, Lee SO (2021). Association of herpes zoster with dementia and effect of antiviral therapy on dementia: a population-based cohort study. Eur Arch Psychiatry Clin Neurosci.

[118] Chen VCH, Wu SI, Huang KY, Yang YH, Kuo TY, Liang HY (2018). Herpes zoster and dementia: a nationwide population-based cohort study. J Clin Psychiatry.

[119] Torniainen-Holm M, Suvisaari J, Lindgren M, Härkänen T, Dickerson F, Yolken RH (2018). Association of cytomegalovirus and Epstein-Barr virus with cognitive functioning and risk of dementia in the general population: 11-year follow-up study. Brain Behav Immun.

[120] Lee KH, Kwon DE, Do Han K, La Y, Han SH (2020). Association between cytomegalovirus end-organ diseases and moderate-to-severe dementia: a population-based cohort study. BMC Neurol.

[121] Wennberg AM, Maher BS, Rabinowitz JA, Holingue C, Felder WR, Wells JL (2023). Association of common infections with cognitive performance in the Baltimore Epidemiologic Catchment Area study follow-up. Alzheimer’s Dementia.

[122] Gale SD, Erickson LD, Brown BL, Hedges DW (2022). Cytomegalovirus is not associated with cognitive function in UK adults aged 40 to 70 years. Psychiatry Res.

[123] Linard M, Letenneur L, Garrigue I, Doize A, Dartigues J, Helmer C (2020). Interaction between APOE4 and herpes simplex virus type 1 in Alzheimer’s disease. Alzheimer’s Dementia.

[124] Linard M, Bezin J, Hucteau E, Joly P, Garrigue I, Dartigues JF (2022). Antiherpetic drugs: a potential way to prevent Alzheimer’s disease?. Alzheimers Res Ther..

[125] Lopatko Lindman K, Weidung B, Olsson J, Josefsson M, Kok E, Johansson A (2019). A genetic signature including apolipoprotein Eε4 potentiates the risk of herpes simplex–associated Alzheimer’s disease. Alzheimer’s Dementia.

[126] Barnes LL, Capuano AW, Aiello AE, Turner AD, Yolken RH, Torrey EF (2015). Cytomegalovirus infection and risk of Alzheimer disease in older black and white individuals. J Infect Dis.

[127] Camacho-Soto A, Faust I, Racette BA, Clifford DB, Checkoway H, Searles NS (2020). Herpesvirus infections and risk of Parkinson’s disease. Neurodegener Dis.

[128] Agostini S, Mancuso R, Costa AS, Citterio LA, Guerini FR, Meloni M (2021). A possible role for HSV-1-specific humoral response and PILRA rs1859788 polymorphism in the pathogenesis of Parkinson’s disease. Vaccines (Basel).

[129] Cheng CM, Bai YM, Tsai CF, Tsai SJ, Wu YH, Pan TL (2020). Risk of Parkinson’s disease among patients with herpes zoster: a nationwide longitudinal study. CNS Spectr.

[130] Lai SW, Lin CH, Lin HF, Lin CL, Lin CC, Liao KF (2017). Herpes zoster correlates with increased risk of Parkinson’s disease in older people. Medicine.

[131] Marttila RJ, Rinne UK (1978). Herpes simplex virus antibodies in patients with Parkinson’s disease. J Neurol Sci.

[132] Marttila RJ, Kalimo KOK, Ziola BR, Halonen PE, Rinne UK (1978). Herpes simplex virus subunit antibodies in patients with Parkinson’s disease. Arch Neurol.

[133] Marttila RJ, Rinne UK, Tiilikainen A (1982). Virus antibodies in Parkinson’s disease. J Neurol Sci.

[134] Bjornevik K, Cortese M, Healy BC, Kuhle J, Mina MJ, Leng Y (2022). Longitudinal analysis reveals high prevalence of Epstein-Barr virus associated with multiple sclerosis. Science.

[135] Khalesi Z, Tamrchi V, Razizadeh MH, Letafati A, Moradi P, Habibi A (2023). Association between human herpesviruses and multiple sclerosis: a systematic review and meta-analysis. Microb Pathog.

[136] Marashi SM, Mostafa A, Shoja Z, Nejati A, Shahmahmoodi S, Mollaei-Kandelous Y (2018). Human herpesvirus 8 DNA detection and variant analysis in patients with multiple sclerosis. Virusdisease.

[137] Opsahl ML, Kennedy PGE (2006). Investigating the presence of human herpesvirus 7 and 8 in multiple sclerosis and normal control brain tissue. J Neurol Sci.

[138] Cermelli C, Vinceti M, Beretti F, Pietrini V, Nacci G, Pietrosemoli P (2002). Risk of sporadic amyotrophic lateral sclerosis associated with seropositivity for herpesviruses and echovirus-7. Eur J Epidemiol.

[139] Bohn B, Lutsey PL, Misialek JR, Walker KA, Brown CH, Hughes TM (2023). Incidence of dementia following hospitalization with infection among adults in the atherosclerosis risk in communities (ARIC) study cohort. JAMA Netw Open.

[140] Fang F, Chen H, Wirdefeldt K, Ronnevi LO, Al-Chalabi A, Peters TL (2011). Infection of the central nervous system, sepsis and amyotrophic lateral sclerosis. PLoS ONE.

[141] Itzhaki RF, Lin WR, Shang D, Wilcock GK, Faragher B, Jamieson GA (1997). Herpes simplex virus type 1 in brain and risk of Alzheimer’s disease. The Lancet.

[142] Miller RM, Federoff HJ (2008). Isoform-specific effects of ApoE on HSV immediate early gene expression and establishment of latency. Neurobiol Aging.

[143] Burgos JS, Ramirez C, Sastre I, Valdivieso F (2006). Effect of apolipoprotein E on the cerebral load of latent herpes simplex virus type 1 DNA. J Virol.

[144] Koelle DM, Magaret A, Warren T, Schellenberg GD, Wald A (2010). APOE genotype is associated with oral herpetic lesions but not genital or oral herpes simplex virus shedding. Sex Transm Infect.

[145] Cairns DM, Itzhaki RF, Kaplan DL (2022). Potential involvement of varicella zoster virus in Alzheimer’s disease via reactivation of quiescent herpes simplex virus type 1. J Alzheimer’s Dis.

[146] Cairns DM, Rouleau N, Parker RN, Walsh KG, Gehrke L, Kaplan DL (2020). A 3D human brain–like tissue model of herpes-induced Alzheimer’s disease. Sci Adv.

[147] Qiao H, Zhao W, Guo M, Zhu L, Chen T, Wang J (2022). Cerebral organoids for modeling of HSV-1-induced-amyloid β associated neuropathology and phenotypic rescue. Int J Mol Sci.

[148] D’Aiuto L, Bloom DC, Naciri JN, Smith A, Edwards TG, McClain L (2019). Modeling herpes simplex virus 1 infections in human central nervous system neuronal cells using two- and three-dimensional cultures derived from induced pluripotent stem cells. J Virol.

[149] Fruhwürth S, Reinert LS, Öberg C, Sakr M, Henricsson M, Zetterberg H (2023). TREM2 is down-regulated by HSV1 in microglia and involved in antiviral defense in the brain. Sci Adv.

[150] Wang HC, Zhang QX, Zhao J, Wei NN (2022). Molecular docking and molecular dynamics simulations studies on the protective and pathogenic roles of the amyloid-β peptide between herpesvirus infection and Alzheimer’s disease. J Mol Graph Model.

[151] Zhu L, Hu C, Mei Y, Zhu M, Ye T (2024). Effect of varicella-zoster virus infection and antiviral treatment on the risk for dementia: a meta-analysis of observational studies. Brain Behav.

[152] Tunnicliffe L, Weil RS, Breuer J, Rodriguez-Barradas MC, Smeeth L, Rentsch CT (2024). Herpes zoster and risk of incident Parkinson’s disease in US veterans: a matched cohort study. Mov Disord.

[153] Itzhaki RF (2023). COVID-19 and Alzheimer’s disease: what is the connection?. J Alzheimer’s Dis.

[154] Le Balc’h P, Pinceaux K, Pronier C, Seguin P, Tadié JM, Reizine F (2020). Herpes simplex virus and cytomegalovirus reactivations among severe COVID-19 patients. Crit Care.

[155] Simonnet A, Engelmann I, Moreau AS, Garcia B, Six S, El Kalioubie A (2021). High incidence of Epstein-Barr virus, cytomegalovirus, and human-herpes virus-6 reactivations in critically ill patients with COVID-19. Infect Dis Now.

[156] Zhang N, Zuo Y, Jiang L, Peng Y, Huang X, Zuo L (2022). Epstein-barr virus and neurological diseases. Front Mol Biosci.

[157] Carbone I, Lazzarotto T, Ianni M, Porcellini E, Forti P, Masliah E (2014). Herpes virus in Alzheimer’s disease: relation to progression of the disease. Neurobiol Aging.

[158] Ascherio A, Munger KL. 99th Dahlem Conference on Infection, Inflammation and Chronic Inflammatory Disorders: Epstein–Barr virus and multiple sclerosis: epidemiological evidence. Clin Exp Immunol. 2010;160(1):120–4. 10.1111/j.1365-2249.2010.04121.x PMC2841845 20415861

[159] Favre-Kontula L, Rolland A, Bernasconi L, Karmirantzou M, Power C, Antonsson B (2008). GlialCAM, an immunoglobulin-like cell adhesion molecule is expressed in glial cells of the central nervous system. Glia.

[160] Menegatti J, Schub D, Schäfer M, Grässer FA, Ruprecht K (2021). HLA-DRB1*15:01 is a co-receptor for Epstein-Barr virus, linking genetic and environmental risk factors for multiple sclerosis. Eur J Immunol.

[161] Li Q, Cohen JI (2019). Epstein-barr virus and the human leukocyte antigen complex. Curr Clin Microbiol Rep.

[162] Yuan L, Deng C, Xue W, He Y, Wang T, Zhang J (2021). Association between HLA alleles and Epstein-Barr virus Zta-IgA serological status in healthy males from southern China. J Gene Med.

[163] Keane JT, Afrasiabi A, Schibeci SD, Swaminathan S, Parnell GP, Booth DR (2021). The interaction of Epstein-Barr virus encoded transcription factor EBNA2 with multiple sclerosis risk loci is dependent on the risk genotype. EBioMedicine.

[164] Gérard HC, Wildt KL, Whittum-Hudson JA, Lai Z, Ager J, Hudson AP (2005). The load of Chlamydia pneumoniae in the Alzheimer’s brain varies with APOE genotype. Microb Pathog.

[165] Feng YK, Wu QL, Peng YW, Liang FY, You HJ, Feng YW (2020). Oral *P. gingivalis* impairs gut permeability and mediates immune responses associated with neurodegeneration in LRRK2 R1441G mice. J Neuroinflammation.

[166] Xue YC, Liu H, Mohamud Y, Bahreyni A, Zhang J, Cashman NR (2022). Sublethal enteroviral infection exacerbates disease progression in an ALS mouse model. J Neuroinflammation.

[167] Young-Xu Y, Powell EI, Zwain GM, Yazdi MT, Gui J, Shiner B (2021). Symptomatic herpes simplex virus infection and risk of dementia in US veterans: a cohort study. Neurotherapeutics.

[168] Schnier C, Janbek J, Williams L, Wilkinson T, Laursen TM, Waldemar G (2021). Antiherpetic medication and incident dementia: observational cohort studies in four countries. Eur J Neurol.

[169] ClinicalTrials.gov ID. NCT03282916.

[170] Devanand DP, Andrews H, Kreisl WC, Razlighi Q, Gershon A, Stern Y (2020). Antiviral therapy: valacyclovir treatment of Alzheimer’s disease (VALAD) Trial: protocol for a randomised, double-blind, placebo-controlled, treatment trial. BMJ Open.

[171] Xue YC, Feuer R, Cashman N, Luo H (2018). Enteroviral infection: the forgotten link to amyotrophic lateral sclerosis?. Front Mol Neurosci.

[172] Ramlow J, Alexander M, LaPorte R, Kaufmann C, Kuller L (1992). Epidemiology of the post-polio syndrome. Am J Epidemiol.

[173] Li Hi Shing S, Chipika RH, Finegan E, Murray D, Hardiman O, Bede P. Post-polio Syndrome: More Than Just a Lower Motor Neuron Disease. Front Neurol. 2019;10. 10.3389/fneur.2019.00773 PMC6646725 31379723

[174] Berger MM, Kopp N, Vital C, Redl B, Aymard M, Lina B (2000). Detection and cellular localization of enterovirus RNA sequences in spinal cord of patients with ALS. Neurology.

[175] Vandenberghe N, Leveque N, Corcia P, Brunaud-Danel V, Salort-Campana E, Besson G (2010). Cerebrospinal fluid detection of enterovirus genome in ALS: a study of 242 patients and 354 controls. Amyotroph Lateral Scler.

[176] Giraud P, Beaulieux F, Ono S, Shimizu N, Chazot G, Lina B (2001). Detection of enteroviral sequences from frozen spinal cord samples of Japanese ALS patients. Neurology.

[177] Woodall CJ, Riding MH, Graham DI, Clements GB (1994). Sequences specific for enterovirus detected in spinal cord from patients with motor neurone disease. BMJ.

[178] Nix WA, Berger MM, Oberste MS, Brooks BR, McKenna-Yasek DM, Brown RH (2004). Failure to detect enterovirus in the spinal cord of ALS patients using a sensitive RT-PCR method. Neurology.

[179] Walker MP, Schlaberg R, Hays AP, Bowser R, Lipkin WI (2001). Absence of echovirus sequences in brain and spinal cord of amyotrophic lateral sclerosis patients. Ann Neurol.

[180] Swanson NR, Fox SA, Mastaglia FL (1995). Search for persistent infection with poliovirus or other enteroviruses in amyotrophic lateral sclerosis-motor neurone disease. Neuromusc Disord..

[181] Kuusisto H, Hyöty H, Kares S, Kinnunen E, Saarelainen M, Elovaara I (2005). Enteroviruses and the risk of MS in the Finnish Twin Cohort. Eur J Neurol.

[182] Perlejewski K, Bukowska-Ośko I, Rydzanicz M, Dzieciątkowski T, Zakrzewska-Pniewska B, Podlecka-Piętowska A (2020). Search for viral agents in cerebrospinal fluid in patients with multiple sclerosis using real-time PCR and metagenomics. PLoS ONE.

[183] Glaubius R, Kothegal N, Birhanu S, Jonnalagadda S, Mahiane SG, Johnson LF (2021). Disease progression and mortality with untreated HIV infection: evidence synthesis of HIV seroconverter cohorts, antiretroviral treatment clinical cohorts and population-based survey data. J Int AIDS Soc.

[184] Collazos J (2003). Opportunistic infections of the CNS in patients with AIDS: diagnosis and management. CNS Drugs.

[185] Maschke M, Kastrup O, Esser S, Ross B, Hengge U, Hufnagel A (2000). Incidence and prevalence of neurological disorders associated with HIV since the introduction of highly active antiretroviral therapy (HAART). J Neurol Neurosurg Psychiatry.

[186] Low A, Gavriilidis G, Larke N, Lajoie MR, Drouin O, Stover J (2016). Incidence of opportunistic infections and the impact of antiretroviral therapy among hiv-infected adults in low- and middle-income countries: a systematic review and meta-analysis. Clin Infect Dis.

[187] Itzhaki RF (2021). Overwhelming evidence for a major role for herpes simplex virus type 1 (HSV1) in Alzheimer’s sisease (AD); underwhelming evidence against. Vaccines (Basel).

[188] Buchbinder SP, Katz MH, Hessol NA, Liu JY, O’Malley PM, Underwood R (1992). Herpes zoster and human immunodeficiency virus infection. J Infect Dis.

[189] Heaton RK, Clifford DB, Franklin DR, Woods SP, Ake C, Vaida F (2010). HIV-associated neurocognitive disorders persist in the era of potent antiretroviral therapy: CHARTER Study. Neurology.

[190] Zenebe Y, Necho M, Yimam W, Akele B (2022). Worldwide occurrence of HIV-associated neurocognitive disorders and its associated factors: a systematic review and meta-analysis. Front Psychiatry.

[191] González-Scarano F, Martín-García J (2005). The neuropathogenesis of AIDS. Nat Rev Immunol.

[192] Thompson PM, Dutton RA, Hayashi KM, Toga AW, Lopez OL, Aizenstein HJ (2005). Thinning of the cerebral cortex visualized in HIV/AIDS reflects CD4+ T lymphocyte decline. Proc Natl Acad Sci.

[193] Wiley CA, Achim C (1994). Human immunodeficiency virus encephalitis is the pathological correlate of dementia in acquired immunodeficiency syndrome. Ann Neurol.

[194] Wendelken LA, Jahanshad N, Rosen HJ, Busovaca E, Allen I, Coppola G (2016). ApoE ε4 is associated with cognition, brain integrity, and atrophy in HIV over age 60. JAIDS.

[195] Clifford KM, Samboju V, Cobigo Y, Milanini B, Marx GA, Hellmuth JM (2017). Progressive brain atrophy despite persistent viral suppression in HIV patients older than 60 years. JAIDS.

[196] Everall I, Vaida F, Khanlou N, Lazzaretto D, Achim C, Letendre S (2009). Cliniconeuropathologic correlates of human immunodeficiency virus in the era of antiretroviral therapy. J Neurovirol.

[197] Anthony IC, Ramage SN, Carnie FW, Simmonds P, Bell JE (2005). Influence of HAART on HIV-related CNS disease and neuroinflammation. J Neuropathol Exp Neurol.

[198] Olsson B, Lautner R, Andreasson U, Öhrfelt A, Portelius E, Bjerke M (2016). CSF and blood biomarkers for the diagnosis of Alzheimer’s disease: a systematic review and meta-analysis. Lancet Neurol.

[199] Fields JA, Swinton MK, Soontornniyomkij B, Carson A, Achim CL (2020). Beta amyloid levels in cerebrospinal fluid of HIV-infected people vary by exposure to antiretroviral therapy. AIDS.

[200] Amod F, Holla VV, Ojha R, Pandey S, Yadav R, Pal PK (2023). A review of movement disorders in persons living with HIV. Parkinsonism Relat Disord.

[201] Ahmad K (2001). HIV may underlie ALS-like condition. Lancet Infect Dis.

[202] MacGowan DJL, Scelsa SN, Waldron M (2001). An ALS-like syndrome with new HIV infection and complete response to antiretroviral therapy. Neurology.

[203] Moulignier A, Moulonguet A, Pialoux G, Rozenbaum W (2001). Reversible ALS-like disorder in HIV infection. Neurology.

[204] Verma A, Berger JR (2006). ALS syndrome in patients with HIV-1 infection. J Neurol Sci.

[205] Cone LA, Nazemi R, Cone MO (2002). Reversible ALS-like disorder in HIV infection An ALS-like syndrome with new HIV infection and complete response to antiretroviral therapy. Neurology.

[206] Sinha S, Mathews T, Arunodaya GR, Siddappa NB, Ranga U, Desai A (2004). HIV-1 clade-C-associated “ALS”-like disorder: first report from India. J Neurol Sci.

[207] Anand KS, Wadhwa A, Garg J, Mahajan RK (2014). Amyotrophic lateral sclerosis-like presentation in a HIV-positive patient. JIAPAC.

[208] John AA, Sharma S, Madhusudhan BK, Mahale R, Javali M, Srinivasa R (2015). A rare presentation of reversible ALS in HIV infection. HIV AIDS Rev.

[209] Bowen LN, Tyagi R, Li W, Alfahad T, Smith B, Wright M (2016). HIV-associated motor neuron disease. Neurology.

[210] Satin ZA, Bayat E (2021). ALS-like disorder in three HIV-positive patients: case series. Case Rep Neurol.

[211] Quevedo-Ramirez A, Montenegro-Idrogo JJ, Resurrección-Delgado C, Salazar-Mesones B, Gallardo-Cartagena J, Cornejo-Venegas G (2020). Lateral amyotrophic sclerosis-like onset after combined antiretroviral treatment initiation. IDCases.

[212] Stefanou MI, Krumbholz M, Ziemann U, Kowarik MC (2019). Human immunodeficiency virus and multiple sclerosis: a review of the literature. Neurol Res Pract..

[213] Xu E, Xie Y, Al-Aly Z (2022). Long-term neurologic outcomes of COVID-19. Nat Med.

[214] Douaud G, Lee S, Alfaro-Almagro F, Arthofer C, Wang C, McCarthy P (2022). SARS-CoV-2 is associated with changes in brain structure in UK Biobank. Nature.

[215] Mitra J, Kodavati M, Provasek VE, Rao KS, Mitra S, Hamilton DJ (2022). SARS-CoV-2 and the central nervous system: emerging insights into hemorrhage-associated neurological consequences and therapeutic considerations. Ageing Res Rev.

[216] Safan AS, Imam Y, Khatib MY, Wraidat M Al, Altermanini MM, Al-Mughalles SA, et al. COVID-19-associated neurological sequelae: a case series on cerebral microbleeds and encephalopathy. Qatar Med J. 2024;2023(4). 10.5339/qmj.2023.29 PMC10618109 37920783

[217] Kurki SN, Kantonen J, Kaivola K, Hokkanen L, Mäyränpää MI, Puttonen H (2021). APOE ε4 associates with increased risk of severe COVID-19, cerebral microhaemorrhages and post-COVID mental fatigue: a Finnish biobank, autopsy and clinical study. Acta Neuropathol Commun.

[218] Gkouskou K, Vasilogiannakopoulou T, Andreakos E, Davanos N, Gazouli M, Sanoudou D (2021). COVID-19 enters the expanding network of apolipoprotein E4-related pathologies. Redox Biol.

[219] Matschke J, Lütgehetmann M, Hagel C, Sperhake JP, Schröder AS, Edler C (2020). Neuropathology of patients with COVID-19 in Germany: a post-mortem case series. Lancet Neurol.

[220] Meinhardt J, Radke J, Dittmayer C, Franz J, Thomas C, Mothes R (2021). Olfactory transmucosal SARS-CoV-2 invasion as a port of central nervous system entry in individuals with COVID-19. Nat Neurosci.

[221] Puelles VG, Lütgehetmann M, Lindenmeyer MT, Sperhake JP, Wong MN, Allweiss L (2020). Multiorgan and renal tropism of SARS-CoV-2. N Engl J Med.

[222] Stein SR, Ramelli SC, Grazioli A, Chung JY, Singh M, Yinda CK (2022). SARS-CoV-2 infection and persistence in the human body and brain at autopsy. Nature.

[223] Crunfli F, Carregari VC, Veras FP, Silva LS, Nogueira MH, Antunes ASLM, et al. Morphological, cellular, and molecular basis of brain infection in COVID-19 patients. Proc Natl Acad Sci. 2022;119(35). 10.1073/pnas.2200960119 PMC9436354 35951647

[224] Song E, Zhang C, Israelow B, Lu-Culligan A, Prado AV, Skriabine S (2021). Neuroinvasion of SARS-CoV-2 in human and mouse brain. J Exp Med.

[225] Bauer L, Lendemeijer B, Leijten L, Embregts CWE, Rockx B, Kushner SA (2021). Replication kinetics, cell tropism, and associated immune responses in SARS-CoV-2- and H5N1 virus-infected human induced pluripotent stem cell-derived neural models. mSphere..

[226] Bullen CK (2020). Infectability of human brainsphere neurons suggests neurotropism of SARS-CoV-2*. Altex.

[227] Ramani A, Müller L, Ostermann PN, Gabriel E, Abida-Islam P, Müller-Schiffmann A (2020). SARS-CoV-2 targets neurons of 3D human brain organoids. EMBO J.

[228] Jacob F, Pather SR, Huang WK, Zhang F, Wong SZH, Zhou H (2020). Human pluripotent stem cell-derived neural cells and brain organoids reveal SARS-CoV-2 neurotropism predominates in choroid plexus epithelium. Cell Stem Cell.

[229] Kettunen P, Lesnikova A, Räsänen N, Ojha R, Palmunen L, Laakso M (2023). SARS-CoV-2 infection of human neurons is TMPRSS2 independent, requires endosomal cell entry, and can be blocked by inhibitors of host phosphoinositol-5 kinase. J Virol.

[230] Samudyata OAO, Malwade S, Rufino de Sousa N, Goparaju SK, Gracias J, et al. SARS-CoV-2 promotes microglial synapse elimination in human brain organoids. Mol Psychiatry. 2022;27(10):3939–50. 10.1038/s41380-022-01786-2 PMC9533278 36198765

[231] Andrews MG, Mukhtar T, Eze UC, Simoneau CR, Ross J, Parikshak N (2022). Tropism of SARS-CoV-2 for human cortical astrocytes. Proc Natl Acad Sci.

[232] Kong W, Montano M, Corley MJ, Helmy E, Kobayashi H, Kinisu M (2022). Neuropilin-1 mediates SARS-CoV-2 infection of astrocytes in brain organoids, inducing inflammation leading to dysfunction and death of neurons. MBio.

[233] Pellegrini L, Albecka A, Mallery DL, Kellner MJ, Paul D, Carter AP (2020). SARS-CoV-2 infects the brain choroid plexus and disrupts the blood-CSF barrier in human brain organoids. Cell Stem Cell.

[234] Jeong GU, Lyu J, Kim KD, Chung YC, Yoon GY, Lee S, et al. SARS-CoV-2 infection of microglia elicits proinflammatory activation and apoptotic cell death. Microbiol Spectr. 2022;10(3). 10.1128/spectrum.01091-22 PMC9241873 35510852

[235] Schwabenland M, Salié H, Tanevski J, Killmer S, Lago MS, Schlaak AE (2021). Deep spatial profiling of human COVID-19 brains reveals neuroinflammation with distinct microanatomical microglia-T-cell interactions. Immunity.

[236] Greene C, Connolly R, Brennan D, Laffan A, O’Keeffe E, Zaporojan L (2024). Blood–brain barrier disruption and sustained systemic inflammation in individuals with long COVID-associated cognitive impairment. Nat Neurosci.

[237] Albornoz EA, Amarilla AA, Modhiran N, Parker S, Li XX, Wijesundara DK (2023). SARS-CoV-2 drives NLRP3 inflammasome activation in human microglia through spike protein. Mol Psychiatry.

[238] Beckman D, Bonillas A, Diniz GB, Ott S, Roh JW, Elizaldi SR (2022). SARS-CoV-2 infects neurons and induces neuroinflammation in a non-human primate model of COVID-19. Cell Rep.

[239] Wang C, Zhang M, Garcia G, Tian E, Cui Q, Chen X (2021). ApoE-isoform-dependent SARS-CoV-2 neurotropism and cellular response. Cell Stem Cell.

[240] Eberle RJ, Coronado MA, Gering I, Sommerhage S, Korostov K, Stefanski A (2023). Tau protein aggregation associated with SARS-CoV-2 main protease. PLoS ONE.

[241] Di Primio C, Quaranta P, Mignanelli M, Siano G, Bimbati M, Scarlatti A (2023). Severe acute respiratory syndrome coronavirus 2 infection leads to Tau pathological signature in neurons. PNAS Nexus..

[242] Miklossy J, Kis A, Radenovic A, Miller L, Forro L, Martins R (2006). Beta-amyloid deposition and Alzheimer’s type changes induced by *Borrelia spirochetes*. Neurobiol Aging.

[243] Miklossy J, Khalili K, Gern L, Ericson RL, Darekar P, Bolle L (2005). *Borrelia burgdorferi* persists in the brain in chronic lyme neuroborreliosis and may be associated with Alzheimer disease. J Alzheimer’s Dis.

[244] Miklossy J (2011). Alzheimer’s disease - a neurospirochetosis. Analysis of the evidence following Koch’s and Hill’s criteria. J Neuroinflammation.

[245] Gutacker M, Valsangiacomo C, Balmelli T, Bernasconi MV, Bouras C, Piffaretti JC (1998). Arguments against the involvement of *Borrelia burgdorferi* sensu lato in Alzheimer’s disease. Res Microbiol.

[246] Forrester JD, Kugeler KJ, Perea AE, Pastula DM, Mead PS (2015). No geographic correlation between lyme disease and death due to 4 neurodegenerative disorders, United States, 2001–2010. Emerg Infect Dis.

[247] Lukehart SA (1988). Invasion of the central nervous system by treponema pallidum: implications for diagnosis and treatment. Ann Intern Med.

[248] Mehrabian S, Raycheva M, Traykova M, Stankova T, Penev L, Grigorova O (2012). Neurosyphilis with dementia and bilateral hippocampal atrophy on brain magnetic resonance imaging. BMC Neurol.

[249] Rao A, Khan A, Singh K, Anderson DL, Malone ML (2015). Neurosyphilis: an uncommon cause of dementia. J Am Geriatr Soc.

[250] Vargas AP, Carod-Artal FJ, Del Negro MC, Rodrigues MPC (2000). Demência por neurossífilis: evolução clínica e neuropsicológica de um paciente. Arq Neuropsiquiatr.

[251] Cassiani-Miranda CA, Chen X (2020). Neurocognitive disorder due to neurosyphilis: a case report. Rev Colomb Psiquiatr.

[252] Stefani A, Riello M, Rossini F, Mariotto S, Fenzi F, Gambina G (2013). Neurosyphilis manifesting with rapidly progressive dementia: report of three cases. Neurol Sci.

[253] Tatar ZB, Cansiz A, Köksal A, Kurt E (2014). A case of neurosyphilis presenting with dementia and psychiatric symptoms. J Neuropsychiatry Clin Neurosci.

[254] Nitrini R, de Paiva ARB, Takada LT, Brucki SMD (2010). Did you rule out neurosyphilis?. Dement Neuropsychol.

[255] Pavlović DM, Milović AM (1999). Clinical characteristics and therapy of neurosyphilis in patients who are negative for human immunodeficiency virus. Srp Arh Celok Lek.

[256] Mukku S, Safal S, Pritam R, Nashi S, Nagarathna C, PT S, et al. Neurosyphilis presenting as rapidly progressive psychosis & dementia—a forgotten entity. Asian J Psychiatr. 2019 Feb;40:103–6. 10.1016/j.ajp.2019.02.010 30785032

[257] Paviolo JP, Imbach MC, Nocenti ZA, Durand BL. [Rapidly progressive dementia due to neurosyphilis (general paralysis). A treatable case of dementia]. Medicina (B Aires). 2020;80(4):401–4. 32841147

[258] Özselek S, Erdem M, Uzun Ö, Ilıca AT, Özşahin A (2011). A neurosyphilis case presenting with dementia. Dusunen Adam.

[259] Blažeković A, Ozretić D, Habek M, Bilić E, Borovečki F (2018). Neurosyphilis: The shape of a rising threat. Int J Infect Dis.

[260] Luo X, Shi H, Hou L, Zhong X, Chen X, Zhang Y (2015). Different cerebrospinal fluid levels of Alzheimer-type biomarker Aβ42 between general paresis and asymptomatic neurosyphilis. Eur J Neurol.

[261] Ryan C, Shaw P, Shorey P. P120 A case of syphilitic amyotrophic lateral sclerosis (ALS)? In: Posters. BMJ Publishing Group Ltd; 2023. p. A81.2-A82.

[262] el Alaoui-Faris M, Medejel A, al Zemmouri K, Yahyaoui M, Chkili T. [Amyotrophic lateral sclerosis syndrome of syphilitic origin. 5 cases]. Rev Neurol (Paris). 1990;146(1):41–4. 2408129

[263] Chraa M, Mebrouk Y, McCaughey C, Kissani N (2013). Amyotrophic lateral sclerosis mimic syndrome due to neurosyphilis. Amyotroph Lateral Scler Frontotemporal Degener.

[264] Tuk B. Syphilis may be a confounding factor, not a causative agent, in syphilitic ALS. F1000Res. 2016;5:1904. 10.12688/f1000research.9318.1 PMC5081158 27830059

[265] Halperin JJ, Kaplan GP, Brazinsky S, Tsai TF, Cheng T, Ironside A (1990). Immunologic reactivity against *Borrelia burgdorferi* in patients with motor neuron disease. Arch Neurol.

[266] Qureshi M, Bedlack RS, Cudkowicz ME (2009). Lyme disease serology in amyotrophic lateral sclerosis. Muscle Nerve.

[267] Mandell H, Steere AC, Reinhardt BN, Yoshinari N, Munsat TL, Brod SA (1989). Lack of antibodies to *Borrelia burgdorferi* in patients with amyotrophic lateral sclerosis. N Engl J Med.

[268] Steiner G (1954). Morphology of Spirochaeta myelophthora in multiple sclerosis. J Neuropathol Exp Neurol.

[269] Chu CS, Liang CS, Tsai SJ, Bai YM, Su TP, Chen TJ (2022). Bacterial pneumonia and subsequent dementia risk: a nationwide cohort study. Brain Behav Immun.

[270] Tate JA, Snitz BE, Alvarez KA, Nahin RL, Weissfeld LA, Lopez O (2014). Infection hospitalization increases risk of dementia in the elderly*. Crit Care Med.

[271] Gérard HC, Dreses-Werringloer U, Wildt KS, Deka S, Oszust C, Balin BJ (2006). Chlamydophila (Chlamydia) pneumoniae in the Alzheimer’s brain. FEMS Immunol Med Microbiol.

[272] Balin BJ, Gérard HC, Arking EJ, Appelt DM, Branigan PJ, Abrams JT (1998). Identification and localization of Chlamydia pneumoniae in the Alzheimer’s brain. Med Microbiol Immunol.

[273] Järvinen H, Tolppanen AM, Hartikainen S (2023). Risk factors of pneumonia in persons with and without Alzheimer’s disease: a matched cohort study. BMC Geriatr.

[274] Manabe T, Fujikura Y, Mizukami K, Akatsu H, Kudo K (2019). Pneumonia-associated death in patients with dementia: a systematic review and meta-analysis. PLoS ONE.

[275] Chacko A, Delbaz A, Walkden H, Basu S, Armitage CW, Eindorf T (2022). Chlamydia pneumoniae can infect the central nervous system via the olfactory and trigeminal nerves and contributes to Alzheimer’s disease risk. Sci Rep.

[276] Rossignol T, Kelly B, Dobson C, d’Enfert C (2011). Endocytosis-mediated vacuolar accumulation of the human ApoE apolipoprotein-derived ApoEdpL-W antimicrobial peptide contributes to its antifungal activity in *Candida albicans*. Antimicrob Agents Chemother.

[277] Petruk G, Elvén M, Hartman E, Davoudi M, Schmidtchen A, Puthia M (2021). The role of full-length apoE in clearance of Gram-negative bacteria and their endotoxins. J Lipid Res.

[278] Porritt RA, Crother TR (2016). *Chlamydia pneumoniae* infection and inflammatory diseases. For Immunopathol Dis Therap.

[279] Turkel Y, Dag E, Gunes H, Apan T, Yoldas T (2015). Is there a relationship between Parkinson′s disease and *Chlamydia pneumoniae*?. Niger J Clin Pract.

[280] Pisa D, Alonso R, Rábano A, Rodal I, Carrasco L (2015). Different brain regions are infected with fungi in Alzheimer’s disease. Sci Rep.

[281] Alonso R, Pisa D, Rábano A, Carrasco L (2014). Alzheimer’s disease and disseminated mycoses. Eur J Clin Microbiol Infect Dis.

[282] Yashkin A, Akushevich I, Yashin A, Gorbunova G, Ukraintseva S (2022). Fungal infections, use of antifungal agents, and the tisk of Alzheimer’s disease. Innov Aging.

[283] https://data.cms.gov/infographic/medicare-beneficiaries-at-a-glance [Internet]. 2024. https://data.cms.gov/infographic/medicare-beneficiaries-at-a-glance.

[284] Pisa D, Alonso R, Carrasco L (2020). Parkinson’s disease: a comprehensive analysis of fungi and bacteria in brain tissue. Int J Biol Sci.

[285] Alonso R, Pisa D, Fernández-Fernández AM, Rábano A, Carrasco L (2017). Fungal infection in neural tissue of patients with amyotrophic lateral sclerosis. Neurobiol Dis.

[286] Alonso R, Pisa D, Carrasco L (2019). Searching for bacteria in neural tissue from amyotrophic lateral sclerosis. Front Neurosci.

[287] Alonso R, Fernández-Fernández AM, Pisa D, Carrasco L (2018). Multiple sclerosis and mixed microbial infections. Direct identification of fungi and bacteria in nervous tissue. Neurobiol Dis.

[288] Pisa D, Alonso R, Jiménez-Jiménez FJ, Carrasco L (2013). Fungal infection in cerebrospinal fluid from some patients with multiple sclerosis. Eur J Clin Microbiol Infect Dis.

[289] Ramos M, Pisa D, Molina S, Rabano A, Juarranz A, Carrasco L (2008). Fungal infection in patients with multiple sclerosis. Open Mycol J.

[290] Benito-León J, Pisa D, Alonso R, Calleja P, Díaz-Sánchez M, Carrasco L (2010). Association between multiple sclerosis and Candida species: evidence from a case-control study. Eur J Clin Microbiol Infect Dis.

[291] Wu Y, Du S, Johnson JL, Tung HY, Landers CT, Liu Y (2019). Microglia and amyloid precursor protein coordinate control of transient Candida cerebritis with memory deficits. Nat Commun.

[292] Soscia SJ, Kirby JE, Washicosky KJ, Tucker SM, Ingelsson M, Hyman B (2010). The Alzheimer’s disease-associated amyloid β-protein is an antimicrobial peptide. PLoS ONE.

[293] Vonk AG, De Bont N, Netea MG, Demacker PNM, van der Meer JWM, Stalenhoef AFH (2004). Apolipoprotein-E-deficient mice exhibit an increased susceptibility to disseminated candidiasis. Med Mycol.

[294] Purzycki CB, Shain DH (2010). Fungal toxins and multiple sclerosis: a compelling connection. Brain Res Bull.

[295] Fraga-Silva TFC, Mimura LAN, Marchetti CM, Chiuso-Minicucci F, França TGD, Zorzella-Pezavento SFG (2015). Experimental autoimmune encephalomyelitis development is aggravated by candida albicans infection. J Immunol Res.

[296] Patel R, Hossain MA, German N, Al-Ahmad AJ (2018). Gliotoxin penetrates and impairs the integrity of the human blood-brain barrier in vitro. Mycotoxin Res.

[297] Nayeri T, Sarvi S, Sharif M, Daryani A (2021). *Toxoplasma gondii*: a possible etiologic agent for Alzheimer’s disease. Heliyon.

[298] Yang HY, Chien WC, Chung CH, Su RY, Lai CY, Yang CC (2021). Risk of dementia in patients with toxoplasmosis: a nationwide, population-based cohort study in Taiwan. Parasit Vectors.

[299] Nayeri Chegeni T, Sarvi S, Moosazadeh M, Sharif M, Aghayan SA, Amouei A (2019). Is *Toxoplasma gondii* a potential risk factor for Alzheimer’s disease? A systematic review and meta-analysis. Microb Pathog.

[300] Mahmoudvand H, Ziaali N, Ghazvini H, Shojaee S, Keshavarz H, Esmaeilpour K (2016). *Toxoplasma gondii* infection promotes neuroinflammation through cytokine networks and induced hyperalgesia in BALB/c mice. Inflammation.

[301] Brandão GP, Melo MN, Caetano BC, Carneiro CM, Silva LA, Vitor RWA (2011). Susceptibility to re-infection in C57BL/6 mice with recombinant strains of *Toxoplasma gondii*. Exp Parasitol.

[302] Jung BK, Pyo KH, Shin KY, Hwang YS, Lim H, Lee SJ (2012). *Toxoplasma gondii* Infection in the brain inhibits neuronal degeneration and learning and memory impairments in a murine model of Alzheimer’s disease. PLoS ONE.

[303] Möhle L, Israel N, Paarmann K, Krohn M, Pietkiewicz S, Müller A (2016). Chronic *Toxoplasma gondii* infection enhances β-amyloid phagocytosis and clearance by recruited monocytes. Acta Neuropathol Commun.

[304] Cicero CE, Allibrio FE, Giuliano L, Luna J, Preux P, Nicoletti A (2021). *Toxoplasma gondii* and multiple sclerosis: a systematic review and meta-analysis. Eur J Neurol.

[305] Nicoletti A, Cicero CE, Giuliano L, Todaro V, Lo Fermo S, Chisari C (2020). *Toxoplasma gondii* and multiple sclerosis: a population-based case–control study. Sci Rep.

[306] Stascheit F, Paul F, Harms L, Rosche B (2015). *Toxoplasma gondii* seropositivity is negatively associated with multiple sclerosis. J Neuroimmunol.

[307] Koskderelioglu A, Afsar I, Pektas B, Gedizlioglu M (2017). Is *Toxoplasma gondii* infection protective against multiple sclerosis risk?. Mult Scler Relat Disord.

[308] Lima TS, Lodoen MB (2019). Mechanisms of human innate immune evasion by *Toxoplasma gondii*. Front Cell Infect Microbiol.

[309] Zhou Y, Cui C, Ma X, Luo W, Zheng SG, Qiu W (2020). Nuclear factor κB (NF-κB)–mediated inflammation in multiple sclerosis. Front Immunol.

[310] Arellano G, Ottum PA, Reyes LI, Burgos PI, Naves R (2015). Stage-specific role of interferon-gamma in experimental autoimmune encephalomyelitis and multiple sclerosis. Front Immunol.

[311] Nagai K, Goto Y (2022). Parasitomimetics: can we utilize parasite-derived immunomodulatory molecules for interventions to immunological disorders?. Front Immunol.

[312] Zaccone P, Fehervari Z, Phillips JM, Dunne DW, Cooke A (2006). Parasitic worms and inflammatory diseases. Parasite Immunol.

[313] Smatti MK, Cyprian FS, Nasrallah GK, Al Thani AA, Almishal RO, Yassine HM (2019). Viruses and autoimmunity: a review on the potential interaction and molecular mechanisms. Viruses.

[314] Oruç S, Karakaya F, Demirbas H, Çeçen İ, Küsbeci ÖY, Miman Ö (2016). Relationship of *Toxoplasma gondii* exposure with multiple sclerosis. Electron J Gen Med.

[315] Rahnama M, Asgari Q, Petramfar P, Tasa D, Hemati V, Solgi R (2020). The role of *toxoplasma gondii* infection among multiple sclerosis patient compared to ordinary people in south of Iran: a case-control study. Modern Care J.

[316] Shahra M, Keshavarz H, Sahraeian MA, Shojaee S, Heidari A, Alimi R (2023). Associations between *Toxoplasma gondii* infection and multiple sclerosis: a case-control seroprevalence study. Iran J Parasitol.

[317] Sevimligul G, Polat ZA, Gokce SF (2023). *Toxoplasma gondii* and multiple sclerosis: a population-based case-control seroprevalence study, Central Anatolia, Turkey. Mult Scler Relat Disord.

[318] McGee DJ, Lu XH, Disbrow EA (2018). Stomaching the possibility of a pathogenic role for *Helicobacter pylori* in Parkinson’s disease. J Parkinsons Dis.

[319] Tan AH, Mahadeva S, Marras C, Thalha AM, Kiew CK, Yeat CM (2015). *Helicobacter pylori* infection is associated with worse severity of Parkinson’s disease. Parkinsonism Relat Disord.

[320] Çamcı G, Oğuz S (2016). Association between Parkinson’s disease and *Helicobacter Pylori*. J Clin Neurol.

[321] Liu NY, Sun JH, Jiang XF, Li H (2021). *Helicobacter pylori* infection and risk for developing dementia: an evidence-based meta-analysis of case-control and cohort studies. Aging.

[322] Sheu J-J, Lin H-C (2013). Association between multiple sclerosis and chronic periodontitis: a population-based pilot study. Eur J Neurol.

[323] Visentin D, Gobin I, Maglica Ž (2023). Periodontal pathogens and their links to neuroinflammation and neurodegeneration. Microorganisms.

[324] Parra-Cantu C, Zaldivar-Ruenes A, Martinez-Vazquez M, Martinez HR (2021). Prevalence of gastrointestinal symptoms, severity of dysphagia, and their correlation with severity of amyotrophic lateral sclerosis in a Mexican cohort. Neurodegener Dis.

[325] Vogt NM, Kerby RL, Dill-McFarland KA, Harding SJ, Merluzzi AP, Johnson SC (2017). Gut microbiome alterations in Alzheimer’s disease. Sci Rep.

[326] Rowin J, Xia Y, Jung B, Sun J (2017). Gut inflammation and dysbiosis in human motor neuron disease. Physiol Rep.

[327] Ferreiro AL, Choi J, Ryou J, Newcomer EP, Thompson R, Bollinger RM (2023). Gut microbiome composition may be an indicator of preclinical Alzheimer’s disease. Sci Transl Med..

[328] Steenblock DA, Ikrar T, San Antonio AS, Wardaningsih E, Azizi MJ (2018). Amyotrophic lateral sclerosis (ALS) linked to intestinal microbiota dysbiosis & systemic microbial infection in human patients: a cross-sectional clinical study. Int J Neurodegener Dis..

[329] Altieri C, Speranza B, Corbo MR, Sinigaglia M, Bevilacqua A (2023). Gut-microbiota, and multiple sclerosis: background, evidence, and perspectives. Nutrients.

[330] Thirion F, Sellebjerg F, Fan Y, Lyu L, Hansen TH, Pons N (2023). The gut microbiota in multiple sclerosis varies with disease activity. Genome Med.

[331] Cantoni C, Lin Q, Dorsett Y, Ghezzi L, Liu Z, Pan Y (2022). Alterations of host-gut microbiome interactions in multiple sclerosis. EBioMedicine.

[332] Mirza A, Forbes JD, Zhu F, Bernstein CN, Van Domselaar G, Graham M (2020). The multiple sclerosis gut microbiota: a systematic review. Mult Scler Relat Disord.

[333] Zhou X, Baumann R, Gao X, Mendoza M, Singh S, Katz Sand I (2022). Gut microbiome of multiple sclerosis patients and paired household healthy controls reveal associations with disease risk and course. Cell.

[334] Sohrabi M, Sahu B, Kaur H, Hasler WA, Prakash A, Combs CK (2022). Gastrointestinal changes and Alzheimer’s disease. Curr Alzheimer Res.

[335] Park AM, Tsunoda I (2022). *Helicobacter pylori* infection in the stomach induces neuroinflammation: the potential roles of bacterial outer membrane vesicles in an animal model of Alzheimer’s disease. Inflamm Regen.

[336] Ding Y, Ren J, Yu H, Yu W, Zhou Y (2018). *Porphyromonas gingivalis*, a periodontitis causing bacterium, induces memory impairment and age-dependent neuroinflammation in mice. Immun Ageing.

[337] Shapira L, Ayalon S, Brenner T (2002). Effects of *Porphyromonas gingivalis* on the central nervous system: activation of glial cells and exacerbation of experimental autoimmune encephalomyelitis. J Periodontol.

[338] Zhou LJ, Lin WZ, Liu T, Chen BY, Meng XQ, Li YL (2023). Oral pathobionts promote MS-like symptoms in mice. J Dent Res.

[339] Fock E, Parnova R (2023). Mechanisms of blood-brain barrier protection by microbiota-derived short-chain fatty acids. Cells.

[340] Silva YP, Bernardi A, Frozza RL (2020). The role of short-chain fatty acids from gut microbiota in gut-brain communication. Front Endocrinol (Lausanne).

[341] Keshavarzian A, Green SJ, Engen PA, Voigt RM, Naqib A, Forsyth CB (2015). Colonic bacterial composition in Parkinson’s disease. Mov Disord.

[342] Saresella M, Marventano I, Barone M, La Rosa F, Piancone F, Mendozzi L (2020). Alterations in circulating fatty acid are associated with gut microbiota dysbiosis and inflammation in multiple sclerosis. Front Immunol.

[343] Lei S, Li J, Yu J, Li F, Pan Y, Chen X (2023). Porphyromonas gingivalis bacteremia increases the permeability of the blood-brain barrier via the Mfsd2a/Caveolin-1 mediated transcytosis pathway. Int J Oral Sci.

[344] Nonaka S, Kadowaki T, Nakanishi H (2022). Secreted gingipains from *Porphyromonas gingivalis* increase permeability in human cerebral microvascular endothelial cells through intracellular degradation of tight junction proteins. Neurochem Int.

[345] Poole S, Singhrao SK, Kesavalu L, Curtis MA, Crean S (2013). Determining the presence of periodontopathic virulence factors in short-term postmortem Alzheimer’s disease brain tissue. J Alzheimer’s Dis.

[346] Ryder MI (2022). The link between periodontitis and Alzheimer’s disease: reality or yet another association. Curr Oral Health Rep.

[347] Dominy SS, Lynch C, Ermini F, Benedyk M, Marczyk A, Konradi A (2019). Porphyromonas gingivali in Alzheimer’s disease brains: evidence for disease causation and treatment with small-molecule inhibitors. Sci Adv.

[348] Raha D, Broce S, Haditsch U, Rodriguez L, Ermini F, Detke M (2020). COR388, a novel gingipain inhibitor, decreases fragmentation of APOE in the central nervous system of Alzheimer’s disease patients. Alzheimer’s Dementia.

[349] Olsen I, Kell DB, Pretorius E (2020). Is *Porphyromonas gingivalis* involved in Parkinson’s disease?. Eur J Clin Microbiol Infect Dis.

[350] Li D, Ren T, Li H, Liao G, Zhang X (2022). Porphyromonas gingivalis: a key role in Parkinson’s disease with cognitive impairment?. Front Neurol.

[351] Adams B, Nunes JM, Page MJ, Roberts T, Carr J, Nell TA (2019). Parkinson’s disease: a systemic inflammatory disease accompanied by bacterial inflammagens. Front Aging Neurosci.

[352] Costa MJF, de Araújo IDT, da Rocha AL, da Silva RL, dos Santos CP, Borges BCD (2021). Relationship of *Porphyromonas gingivalis* and Alzheimer’s disease: a systematic review of pre-clinical studies. Clin Oral Investig.

[353] Storelli E, Cassina N, Rasini E, Marino F, Cosentino M (2019). Do Th17 lymphocytes and IL-17 contribute to Parkinson’s disease? A systematic review of available evidence. Front Neurol.

[354] Ha JY, Seok J, Kim SJ, Jung HJ, Ryu KY, Nakamura M (2023). Periodontitis promotes bacterial extracellular vesicle-induced neuroinflammation in the brain and trigeminal ganglion. PLoS Pathog.

[355] Noori M, Mahboobi R, Nabavi-Rad A, Jamshidizadeh S, Fakharian F, Yadegar A (2023). *Helicobacter pylori* infection contributes to the expression of Alzheimer’s disease-associated risk factors and neuroinflammation. Heliyon.

[356] Xie J, Cools L, Van Imschoot G, Van Wonterghem E, Pauwels MJ, Vlaeminck I (2023). *Helicobacter pylori*-derived outer membrane vesicles contribute to Alzheimer’s disease pathogenesis via C3–C3aR signalling. J Extracell Vesicles..

[357] Eimer WA, Vijaya Kumar DK, Navalpur Shanmugam NK, Rodriguez AS, Mitchell T, Washicosky KJ (2018). Alzheimer’s disease-associated β-amyloid is rapidly seeded by herpesviridae to protect against brain infection. Neuron.

[358] Bourgade K, Garneau H, Giroux G, Le Page AY, Bocti C, Dupuis G (2015). β-Amyloid peptides display protective activity against the human Alzheimer’s disease-associated herpes simplex virus-1. Biogerontology.

[359] Bocharova O, Pandit NP, Molesworth K, Fisher A, Mychko O, Makarava N (2021). Alzheimer’s disease-associated β-amyloid does not protect against herpes simplex virus 1 infection in the mouse brain. J Biol Chem.

[360] Kobayashi N, Masuda J, Kudoh J, Shimizu N, Yoshida T (2008). Binding sites on tau protein as components for antimicrobial peptides. Biocontrol Sci.

[361] Kanagasingam S, von Ruhland C, Welbury R, Singhrao SK (2022). Antimicrobial, polarizing light, and paired helical filament properties of fragmented tau peptides of selected putative gingipains. J Alzheimer’s Dis.

[362] Alam MM, Yang D, Li XQ, Liu J, Back TC, Trivett A (2022). Alpha synuclein, the culprit in Parkinson disease, is required for normal immune function. Cell Rep.

[363] Liu-Yesucevitz L, Bilgutay A, Zhang YJ, Vanderwyde T, Citro A, Mehta T (2010). Tar DNA binding protein-43 (TDP-43) associates with stress granules: analysis of cultured cells and pathological brain tissue. PLoS ONE.

[364] Sama RRK, Ward CL, Kaushansky LJ, Lemay N, Ishigaki S, Urano F (2013). FUS/TLS assembles into stress granules and is a prosurvival factor during hyperosmolar stress. J Cell Physiol.

[365] McCormick C, Khaperskyy DA (2017). Translation inhibition and stress granules in the antiviral immune response. Nat Rev Immunol.

[366] Fung G, Shi J, Deng H, Hou J, Wang C, Hong A (2015). Cytoplasmic translocation, aggregation, and cleavage of TDP-43 by enteroviral proteases modulate viral pathogenesis. Cell Death Differ.

[367] Xue YC, Ng CS, Mohamud Y, Fung G, Liu H, Bahreyni A (2021). FUS/TLS suppresses enterovirus replication and promotes antiviral innate immune responses. J Virol.

[368] Shelkovnikova TA, An H, Skelt L, Tregoning JS, Humphreys IR, Buchman VL (2019). Antiviral immune response as a trigger of FUS proteinopathy in amyotrophic lateral sclerosis. Cell Rep.

[369] Dunker W, Ye X, Zhao Y, Liu L, Richardson A, Karijolich J (2021). TDP-43 prevents endogenous RNAs from triggering a lethal RIG-I-dependent interferon response. Cell Rep.

[370] Licht-Murava A, Meadows SM, Palaguachi F, Song SC, Jackvony S, Bram Y (2023). Astrocytic TDP-43 dysregulation impairs memory by modulating antiviral pathways and interferon-inducible chemokines. Sci Adv.

[371] Cabrera JR, Rodríguez-Izquierdo I, Jiménez JL, Muñoz-Fernández MÁ (2020). Analysis of ALS-related proteins during herpes simplex virus-2 latent infection. J Neuroinflammation.

[372] Choi SW, Mak TSH, O’Reilly PF (2020). Tutorial: a guide to performing polygenic risk score analyses. Nat Protoc.

[373] Bakaletz LO (2004). Developing animal models for polymicrobial diseases. Nat Rev Microbiol.

[374] Rõlova T, Lehtonen Š, Goldsteins G, Kettunen P, Koistinaho J (2021). Metabolic and immune dysfunction of glia in neurodegenerative disorders: focus on iPSC models. Stem Cells.

[375] Stöberl N, Maguire E, Salis E, Shaw B, Hall-Roberts H (2023). Human iPSC-derived glia models for the study of neuroinflammation. J Neuroinflammation.

[376] Harris WJ, Asselin MC, Hinz R, Parkes LM, Allan S, Schiessl I (2023). In vivo methods for imaging blood–brain barrier function and dysfunction. Eur J Nucl Med Mol Imaging.

[377] Kadry H, Noorani B, Cucullo L (2020). A blood–brain barrier overview on structure, function, impairment, and biomarkers of integrity. Fluids Barriers CNS.

[378] Vigh JP, Kincses A, Ozgür B, Walter FR, Santa-Maria AR, Valkai S (2021). Transendothelial electrical resistance measurement across the blood-brain barrier: a critical review of methods. Micromachines (Basel).

[379] Alves AH da, Nucci MP, Ennes Valle NM do, Missina JM, Mamani JB, Rego GNA, et al. Current overview of induced pluripotent stem cell-based blood-brain barrier-on-a-chip. World J Stem Cells. 2023;15(6):632–53. 10.4252/wjsc.v15.i6.632 PMC10324508 37424947

[380] Moussa C, Hebron M, Huang X, Ahn J, Rissman RA, Aisen PS (2017). Resveratrol regulates neuro-inflammation and induces adaptive immunity in Alzheimer’s disease. J Neuroinflammation.

[381] Zhang Y, Guo H, Fu H (2024). Protective effect of resveratrol combined with levodopa against oxidative damage in dopaminergic neurons. Cell Biochem Biophys.

[382] Chen X, Song X, Zhao X, Zhang Y, Wang Y, Jia R (2022). Insights into the anti-inflammatory and antiviral mechanisms of resveratrol. Mediators Inflamm.

[383] Khosropour S, Shahvarooghi E, Rezaeizadeh H, Esmaeelzadeh M (2024). Curcumin and its semisynthetic derivative F-curcumin ameliorate the expression of cytokines in autoimmune encephalomyelitis mouse models of multiple sclerosis. Iran J Allergy Asthma Immunol.

[384] Giacobbe J, Benoiton B, Zunszain P, Pariante CM, Borsini A (2020). The anti-inflammatory role of omega-3 polyunsaturated fatty acids metabolites in pre-clinical models of psychiatric, neurodegenerative, and neurological disorders. Front Psychiatry.

[385] Gong L, Zhu T, Chen C, Xia N, Yao Y, Ding J (2022). Miconazole exerts disease-modifying effects during epilepsy by suppressing neuroinflammation via NF-κB pathway and iNOS production. Neurobiol Dis.

